# COGNAT: a web server for comparative analysis of genomic neighborhoods

**DOI:** 10.1186/s13062-017-0196-z

**Published:** 2017-11-22

**Authors:** Olesya I. Klimchuk, Kirill A. Konovalov, Vadim V. Perekhvatov, Konstantin V. Skulachev, Daria V. Dibrova, Armen Y. Mulkidjanian

**Affiliations:** 1 School of Bioengineering and Bioinformatics, Lomonosov Moscow State University, 119991 Moscow, Russia; 2 School of Chemistry, Lomonosov Moscow State University, 119991 Moscow, Russia; 3 Belozersky Institute of Physico-Chemical Biology, Lomonosov Moscow State University, 119991 Moscow, Russia; 4 Department of Physics, Osnabrueck University, 49069, Osnabrueck, Germany

**Keywords:** Clusters of orthologous groups of proteins, Operon, Phylogenomic analysis, Comparative genomics, Orthologs, Paralogs, NADH:Quinone oxidoreductase of type 1

## Abstract

**Background:**

In prokaryotic genomes, functionally coupled genes can be organized in conserved gene clusters enabling their coordinated regulation. Such clusters could contain one or several operons, which are groups of co-transcribed genes. Those genes that evolved from a common ancestral gene by speciation (i.e. orthologs) are expected to have similar genomic neighborhoods in different organisms, whereas those copies of the gene that are responsible for dissimilar functions (i.e. paralogs) could be found in dissimilar genomic contexts. Comparative analysis of genomic neighborhoods facilitates the prediction of co-regulated genes and helps to discern different functions in large protein families.

**Aim:**

We intended, building on the attribution of gene sequences to the clusters of orthologous groups of proteins (COGs), to provide a method for visualization and comparative analysis of genomic neighborhoods of evolutionary related genes, as well as a respective web server.

**Results:**

Here we introduce the COmparative Gene Neighborhoods Analysis Tool (COGNAT), a web server for comparative analysis of genomic neighborhoods. The tool is based on the COG database, as well as the Pfam protein families database. As an example, we show the utility of COGNAT in identifying a new type of membrane protein complex that is formed by paralog(s) of one of the membrane subunits of the NADH:quinone oxidoreductase of type 1 (COG1009) and a cytoplasmic protein of unknown function (COG3002).

**Reviewers:**

This article was reviewed by Drs. Igor Zhulin, Uri Gophna and Igor Rogozin.

**Electronic supplementary material:**

The online version of this article (10.1186/s13062-017-0196-z) contains supplementary material, which is available to authorized users.

## Implementation

The COGNAT web server enables comparative analysis of genomic neighborhoods of evolutionary related genes from the manually curated representative set of 711 completely sequenced prokaryotic genomes, on which the most recent release of the Clusters of Orthologous Groups of proteins (COGs) database (http://www.ncbi.nlm.nih.gov/COG/) [1] is based.

### Annotating proteins from the representative set of prokaryotic genomes

**Fig. 1:**
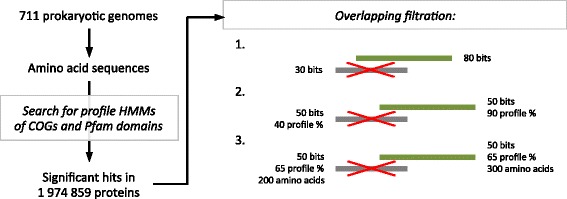
A scheme of obtaining annotations of COGs and Pfam domains for proteins from 711 prokaryotic genomes (see the main text for details)

We obtained annotations of COGs and Pfam domains for proteins encoded in the representative set of 711 prokaryotic genomes by searching profile hidden Markov models (HMMs) against the set of amino acid sequences with the HMMer software (http://hmmer.org/ [2]) (Fig. 1). Namely, the profile HMMs from the release 30.0 of the Pfam protein families database [3] and the profile HMMs of COGs [1] were used. The set of profile HMMs of COGs was obtained as described in [4] and contained 4534 entries in total.

The search with the profile HMM against the set of amino acid sequences yields a list of regions in any sequence from the set, whose similarity to the profile HMM appears to be non-random (regions are termed as “hits”). We ignored a hit if its score was less than 25.0 bits or if its length was less than 25% of the profile HMM length. Therefore, significant hits were found in 1,974,859 proteins encoded in 711 prokaryotic genomes, and 419,609 proteins did not obtain a valid annotation. In many proteins, several significant Pfam domain hits were found. In order to assign such proteins to a particular architecture of Pfam domains, overlapping hits were filtered according to the following criteria:

- if a lower-scored hit was overlapped by a higher-scored hit for more than 50%, such a lower-scored hit was ignored;
- if two overlapping hits had an equal score, we normalized the length of each hit on the length of the respective profile HMM, and we preferred the hit that had a greater normalized length;
- if two overlapping hits had an equal score and an equal normalized length, we preferred the longer hit.

Some proteins were attributed to several COGs; if the respective hits overlapped, they were filtered according to the criteria described above.

### Visualizing genomic neighborhoods

Each gene is represented in the COGNAT as an arrow, and each intergenic region is represented as a single line. The direction of the arrow indicates on which DNA strand the gene is encoded relatively to the target gene which is always represented by an arrow that points to the right. The lengths of each arrow and each line correspond to the length of the respective gene and the respective intergenic region. An arrow can be colored in accordance with the annotation of the gene product; specific colors are assigned to each COG and each Pfam domain. In particular, all the Pfam domains that belong to the same Pfam clan [3] are colored identically.

### User interface of the COGNAT

**Fig. 2:**
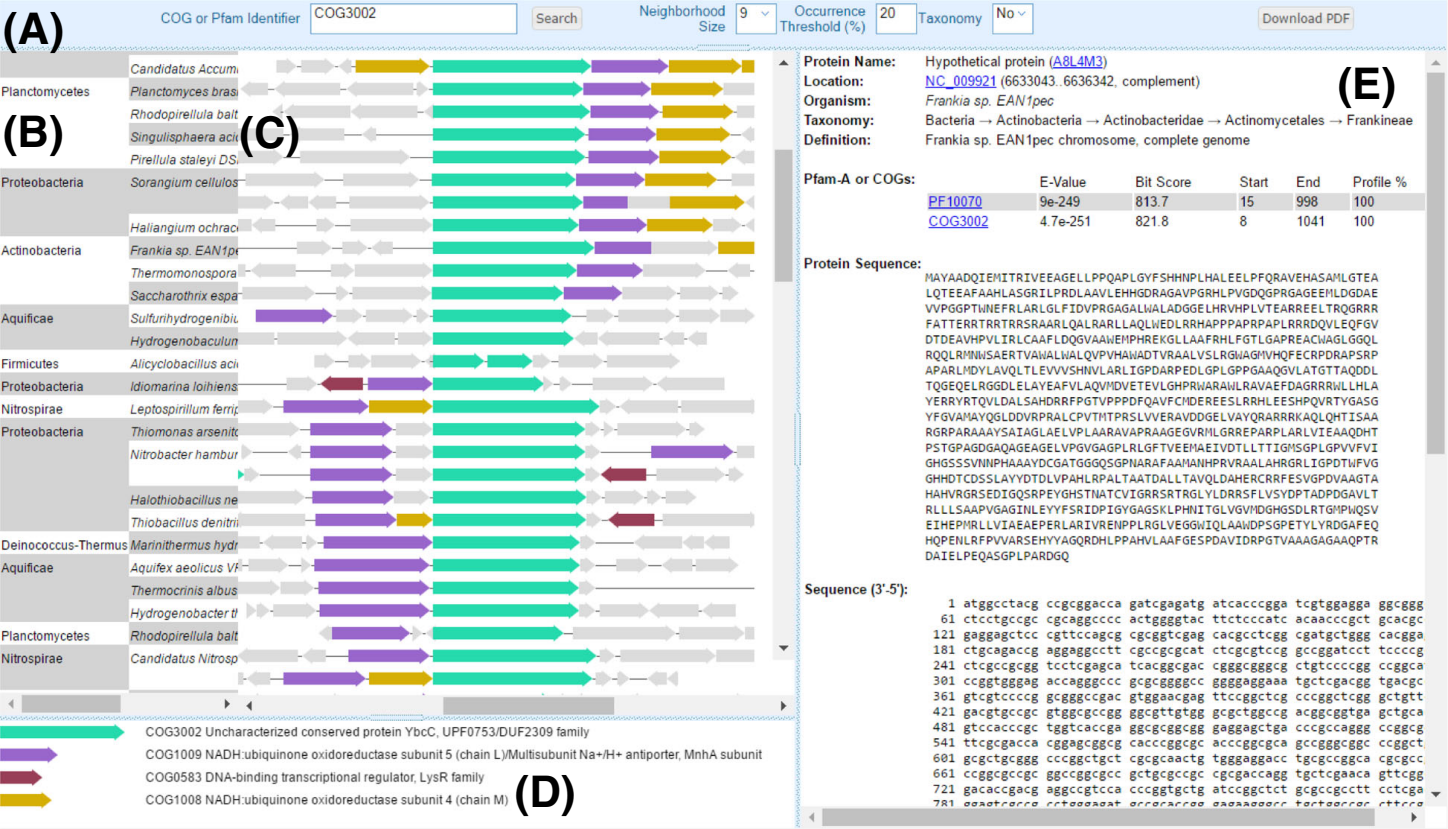
A screenshot of the COGNAT main window running in the COG mode. (**a**), Request field and options panel; (**b**), two high-level taxonomy units for each nucleotide record; (**c**), genetic neighborhoods where genes are colored according to the panel (**d**); (**d**), the color code and domain annotation for the panel (**c**); (**e**), a detailed annotation for a clicked gene

The main panel of the COGNAT is shown in Fig. 2. A user is expected to specify a COG identifier (e.g. COG0001) or a Pfam domain accession number (e.g. PF00001) in order to run the server. In the case of a COG identifier, genomic neighborhoods of all genes, whose products were attributed to the particular COG by Galperin and colleagues [1], are visualized. In the case of a Pfam domain accession number, genomic neighborhoods of all genes, whose products contain the Pfam domain (according to the annotation procedure described above), are depicted.

By default, genomic neighborhoods of the target genes are sorted in accordance with the multiple sequence alignment of the respective amino acid sequences built with the MUSCLE software (default parameters are used, except the number of iterations being set to two) [5]. This type of sorting allows to group genomic neighborhoods of closely related genes, which is particularly interesting for predicting new protein complexes. Genomic neighborhoods of the target genes can also be sorted in accordance with the prokaryotic taxonomy; if an organism has several target genes, their genomic neighborhoods are listed under each other. This type of sorting is useful in estimating the overall distribution of the members of a COG or a Pfam domain among the representative set of 711 prokaryotic genomes and could be helpful for the analysis of paralogous genes in particular genomes.

The size of a neighborhood could be chosen in the range from 3 to 15 genes. Neighboring genes are automatically colored in accordance with the occurrence-percentage threshold (from 1% to 100%). For example, if the threshold value was set to 20%, and the server is running for the COG3002, then genomic neighborhoods of 115 target genes are visualized. In order not to miss possible important neighbors, we suggest to use the minimal 1% threshold first, and then raise it if necessary. The genes of the proteins that are attributed to the COG1009 occur within the neighborhoods more than 22 times, even if the size of a neighborhood was set to 3 genes, and therefore are automatically colored. This option is useful for identification of co-localized genes. A color legend is given below the main figure and provides a list of domain descriptions taken from the original COG or Pfam database [1, 3].

Annotations of each gene and each intergenic region of the neighborhood are available by clicking on an arrow or a line, respectively. An annotation of a gene comprises a description of the protein, references to the UniProt database [6], the genomic coordinates of the gene, the taxonomy of the organism, the annotations of both the COGs and Pfam domains, the protein sequence, and the gene sequence. A list of hits for both COGs and Pfam domains with their parameters (coordinates, scores, e-values) is also shown for each gene. An annotation of an intergenic region comprises the genomic coordinates of the intergenic region, the taxonomy of the organism, the nucleotide sequence, and the reverse complement sequence.

The main output of the COGNAT can be downloaded to the local computer as a PDF file.

### Comparison with other web-based servers for comparative genomics of prokaryotes

The possibility to compare gene neighborhoods in prokaryotic genomes is incorporated in such web servers as MicrobesOnline [7], PSAT [8], and STRING [9]. In case of MicrobesOnline [7], a phylogenetic tree-based genome browser visualizes genomic neighborhoods for homologous genes that are searched by using the FastBLAST routine [10]. The maximal number of genomic neighborhoods is 400, so that distant homologs are either ignored or could be shown in the form of clusters. In contrast to this approach, COGNAT does not cluster sequences and is not limited to the number of hits, so that all proteins attributed to a particular COG or Pfam domain are shown (this is specifically important for big protein families which frequently occur in large taxonomic groups, e.g. Proteobacteria). This feature might be helpful upon studies where the peculiarities of genomic contexts should be inspected on a case-to-case basis.

The PSAT web server [8] identifies, for each gene in a reference genome, the three top-scored hits in each comparison genome by launching the protein BLAST routine [11]; the genomic neighborhoods are clustered in accordance with the number of genes surrounding a given homolog in a conserved order. For proteins that belong to multiCOG superfamilies, approaches that are based on the BLAST search would not allow to analyze a particular COG. By contrast, COGNAT starts from a pre-defined set of proteins (either provided by the COG database [1] or obtainable, for each Pfam domain, by using the procedure described in [4]). Our approach enables the analysis of a complete COG, including its truncated or “weak” members, which are likely to get lost during a cut-off procedure of the PSAT web server. Such COG members could be important as non-standard versions of a protein, e.g. upon establishing relatedness to other COG(s).

The web server of the STRING database [9] is focused on the evidences of functional associations between proteins; it does not provide either the sequences of genes and intergenic regions or the detailed annotation of COGs and Pfam domains in a protein.

### Availability and requirements

- Project name: COGNAT
- Project home page: https://depo.msu.ru/module/cognat
- Operating system(s): Platform independent (web server)
- Programming language: C++, Java, JavaScript, HTML5
- Other requirements: Java SE 1.8 or higher, Tomcat 7.0 or higher
- License: GNU GPL
- Any restrictions to use by non-academics: none declared

## Main text

### Application example

**Fig. 3:**
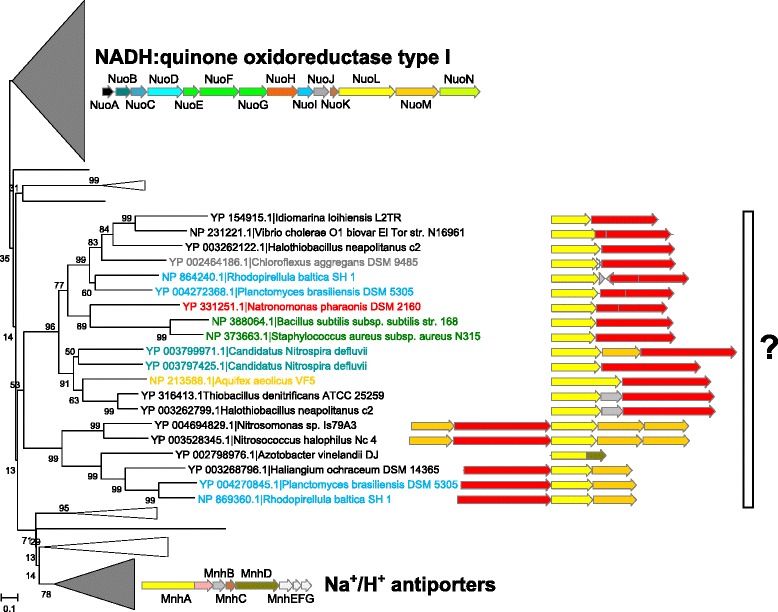
Genomic neighborhoods for the clades of predicted proteins belonging to the COG1009 (NADH:ubiquinone oxidoreductase of type 1, NuoL subunit, and Na^+^/H^+^ antiporter, MnhA subunit). Typical genomic neighborhoods are shown for the top and bottom clades of the tree that contain sequences of well-studied proteins. A set of 177 proteins belonging to a reduced but representative set of 124 genomes was aligned with the MUSCLE software [5]. The multiple alignment was verified manually and conserved blocks (total 238 positions) were used for the phylogenetic tree construction with the MEGA 7 software [17]. Bootstrap support values calculated from 100 iterations are shown on the branches. Red arrows correspond to the proteins that belong to COG3002

The preliminary version of the COGNAT software has already helped us to identify a new type of membrane energy-converting complex, a tentative sodium-translocating decarboxylating oxidoreductase [12], and to specify evolutionary relations between the cytochrome *bc* complexes [13]. Here, we have applied the COGNAT web server to the COG1009 that comprises two homologous protein families: the MnhA subunit of Na^+^/H^+^ antiporters [14] and the membrane subunit NuoL of NADH:quinone oxidoreductases of type 1 (NDH-1) [15, 16]. In both cases, proteins appear to be involved in the transmembrane translocation of protons and/or sodium ions (see [14–16] for reviews). The COGNAT data were mapped on the phylogenetic tree of these proteins (Fig. 3, see the figure caption for the tree construction details). In addition to the previously described families of NDH-1 and Na^+^/H^+^ antiporters, we identified a strongly supported clade with an operon that may code for a new membrane protein complex built of membrane-embedded homolog(s) of NuoL (COG1009, colored yellow), and a water-soluble protein of unknown function (COG3002, colored red). The gene of this water-soluble protein could be found in diverse bacteria; in *Bacillus subtilis* it was named *YbcC*, so that hereafter we use this name*.* In archaea, the complex could be found only in *Haloarchaea*, which are known to contain a plethora of bacterial genes derived via the lateral gene transfer [7]. The *ybcC* gene is always found alongside the homolog of the *nuoL* gene (see (Additional file 1: Figure S1) for the phylogenetic tree of the COG3002 proteins with their genetic neighborhoods visualized with the help of the COGNAT software).

Our analysis indicates that the operon structures of the *ybcC* genes show systematic variations:

- if the *ybcC* gene is coded downstream of the homolog of the *nuoL* gene, either the gene of a DNA-binding protein from the LysR family (COG0583) is frequently found in a reverse direction at the 5′ end of the predicted operon (clade A in Additional file 1: Figure S1), or the gene of a small nitrogen regulatory protein PII (COG0347) is found at the 3′ end of the predicted operon (clade B in Additional file 1: Figure S1).
- if the *ybcC* gene is coded upstream of the homolog of the *nuoL* gene, a genetic neighborhood frequently contains genes of additional membrane subunits, namely COG0659 (sulfate permease or a related transporter) and COG1008 (NuoM, another subunit of NDH-1) (clade C in Additional file 1: Figure S1).

We suggest that the here identified operons code for membrane energy-converting enzyme complexes of a novel type. Proteins from COG3002 contain many conserved polar residues (see Additional file 2: Figure S2, for the multiple alignment), including three cysteine residues; these proteins, however, appear to have an unknown fold, so that we were unable to predict their function (the corresponding Pfam domain PF10070/DUF2309 does not belong to any clan, and distantly related COGs are also missing). The apparent affiliation of the YbcC proteins with homologs of membrane ion-translocating proteins prompted us to suggest that the products of the *ybcC* gene and its homologs may catalyze exergonic reactions, e.g. oxidation or cleavage of some substrate(s). Such exergonic reactions could yield enough energy to translocate proton(s) or sodium ion(s) out of the cell, from the negatively charged side of the membrane to its positively charged side, by a coupled homolog of the NuoL subunit, thus generating the respective ion gradient. The number of genes encoding the membrane subunits in an operon might be related to the number of translocated ions: the operons with a single gene of a membrane subunit could be responsible for translocation of only one ion, whereas the operons with multiple genes of membrane subunits could be responsible for translocation of several ions, by analogy with the NDH-1 and its homologs [15].

## Reviewers’ comments

We thank the reviewers for their valuable comments and helpful suggestions that helped us to improve the manuscript.

### Reviewer’s report 1: Dr. Igor Zhulin, Oak Ridge National Laboratory

**Reviewer 1**: Klimchuk et al. describe COGNAT, a new tool for comparative analysis of gene neighborhoods. This type of analysis is an important component of comparative genomics of bacteria and archaea, which leads to a discovery of novel components of metabolic and signaling pathways. The tool allows exploring the gene neighborhoods using COG or Pfam identifiers. Consequently, if a protein of interest is well defined in either COG or Pfam terms, the tool will be quite useful. The web server has an intuitive interface and it is easy to use. The layout is appealing (a split screen separating gene neighborhood visualization and sequence information) and an easy access to relevant information, e.g. sequence and its identifiers, scores for matching to domain models (both E-value and bit score), taxonomy, etc. The paper is well written and describes the tool in sufficient detail. Comparison with other tools for analysis of gene neighborhoods emphasizing the COGNAT advantages is also provided. Authors demonstrated their application in action by exploring COG1009 neighborhoods. I gave the web server a try by exploring gene neighborhoods for a couple of my favorite genes and it worked out very well.

The obvious limitation of COGNAT is that it uses a relatively small number of genomes that are available in the COG database, but there is no way around this problem without very arduous efforts... I have no concerns about either the server or the paper.

Authors’ response: *We would like to thank the reviewer for his positive comments on the manuscript, as well as for testing our web server. We are concerned about the relatively small number of genomes that are available in the COG database as well. Therefore we are currently working on a desktop version of the program, which would be able to work with genome samples of any size and would have more customizable output options*.

### Reviewer’s report 2: Prof. Uri Gophna, The George S. Wise Faculty of Life Sciences, Tel Aviv University

**Reviewer 2**: This manuscript describes what will surely be is a very useful server, and since its main “competitor” has not been functional for a while, is also urgently required. I have just a few minor comments, see below.

Authors’ response: *We thank the reviewer for his efforts to improve the manuscript quality and readability.*

**Reviewer 2**: Abstract: Operons are by definition co-transcribed as one mRNA (though there can also be internal promoters), while gene clusters can contain multiple transcription start sites, and even divergently transcribed genes. Clusters are functionally important and can contain more than one operon etc., so the distinction should be clearer.

Authors’ response: *We have rephrased the aforementioned sentence to highlight the difference and improve clarity: “In prokaryotic genomes, functionally coupled genes can be organized in conserved gene clusters enabling their coordinated regulation. Such clusters could contain one or several operons, which are groups of co-transcribed genes”.*

**Reviewer 2**: "GOGNAT starts from a pre-defined set of proteins" should be “COGNAT”.

Authors’ response: *The typo has been fixed*.

**Reviewer 2**: Line 152 “Main text” - looks out of place.

Authors’ response: *This section name is given in accordance with the Biology Direct author guidelines for Application Notes.*

**Reviewer 2**: Line 192 - "from the n-side of the membrane to the p-side of the membrane", rephrase to improve clarity as these acronyms are not commonly used.

Authors’ response: *We agree with the Reviewer. We have rephrased the sentence as “from the negatively charged side of the membrane to its positively charged side”*.

### Reviewer’s report 3: Dr. Igor Rogozin, National Center for Biotechnology Information, NLM, NIH

**Reviewer 3**: The paper describes a web server for analysis of genomic neighborhoods. I do not see major methodological problems.

I understand that this is hard to implement but still some approximate estimates of the significance of the occurrence-percentage value will substantially improve the program. I do not insist on this for this paper but this may be an important venue for future developments of the program. This issue is likely to be important for relatively rare (but still functionally important) gene pairs. This statistics can be done using Monte Carlo (e.g. Rogozin et al. Connected gene neighborhoods in prokaryotic genomes. Nucleic Acids Res. 2002 30(10):2212–23).

Authors’ response: *We agree with the Reviewer that the usage of the percent of occurrence as a criterion for important functional interaction is not optimal. We are currently working over the desktop version of our server with many additional options. In this version, we would use several criteria of importance, including the measure suggested by the reviewer. Therefore we are very thankful to the Reviewer for providing us with the respective reference.*

*In addition, in the revised manuscript, we have added the following sentence: “In order not to miss possible important neighbors, we suggest to use the minimal 1% threshold first, and then raise it if necessary”.*

## Additional files

Additional file 1: Figure S1. Phylogenetic tree for the proteins belonging to the COG3002. All 115 proteins from 711 genomes, as available in the COG database, were sampled, with only three truncated sequences being removed. Proteins were aligned with the MUSCLE software [5], conserved blocks' regions containing 384 positions were selected manually. The phylogenetic tree was constructed with the MEGA 7 software [17]. Bootstrap support values calculated from 100 iterations are shown on the branches. A color legend is given below the figure (PDF 143 kb)

Additional file 2: Figure S2. A part of a multiple sequence alignment of proteins belonging to the COG3002 (uncharacterized conserved protein YbcC, UPF0753/DUF2309 family). Only conserved blocks' regions with at least 95% conserved charged residues are shown (see the scheme below for their coordinates in the YbcC protein from *Bacillus subtilis*). Such conserved residues, which could be catalytically important, are marked with the “X” sign in the SITE pseudo-sequence. The multiple alignment was constructed with the MUSCLE software [5] and visualized with the help of the GeneDoc software (DOCX 225 kb)

## Abbreviations

COG: Cluster of Orthologous Groups
HMM: Hidden Markov Model
NDH-1: NADH:quinone oxidoreductase of type 1
