# Supplementary material for: COGNAT: a web server for comparative analysis of genomic neighborhoods
Source: Biol Direct. 2017 Nov 22;12:26. doi: 10.1186/s13062-017-0196-z (PMC5700660; doi:10.1186/s13062-017-0196-z)
Supplement: Supplementary file 1 — Phylogenetic tree for the proteins belonging to the COG3002. All 115 proteins from 711 genomes, as available in the COG database, were sampled, with only three truncated sequences being removed. Proteins were aligned with the MUSCLE software [5], conserved blocks' regions containing 384 positions were selected manually. The phylogenetic tree was constructed with the MEGA 7 software [17]. Bootstrap support values calculated from 100 iterations are shown on the branches. A color legend is given below the figure (PDF 143 kb) [file 13062_2017_196_MOESM1_ESM.pdf]

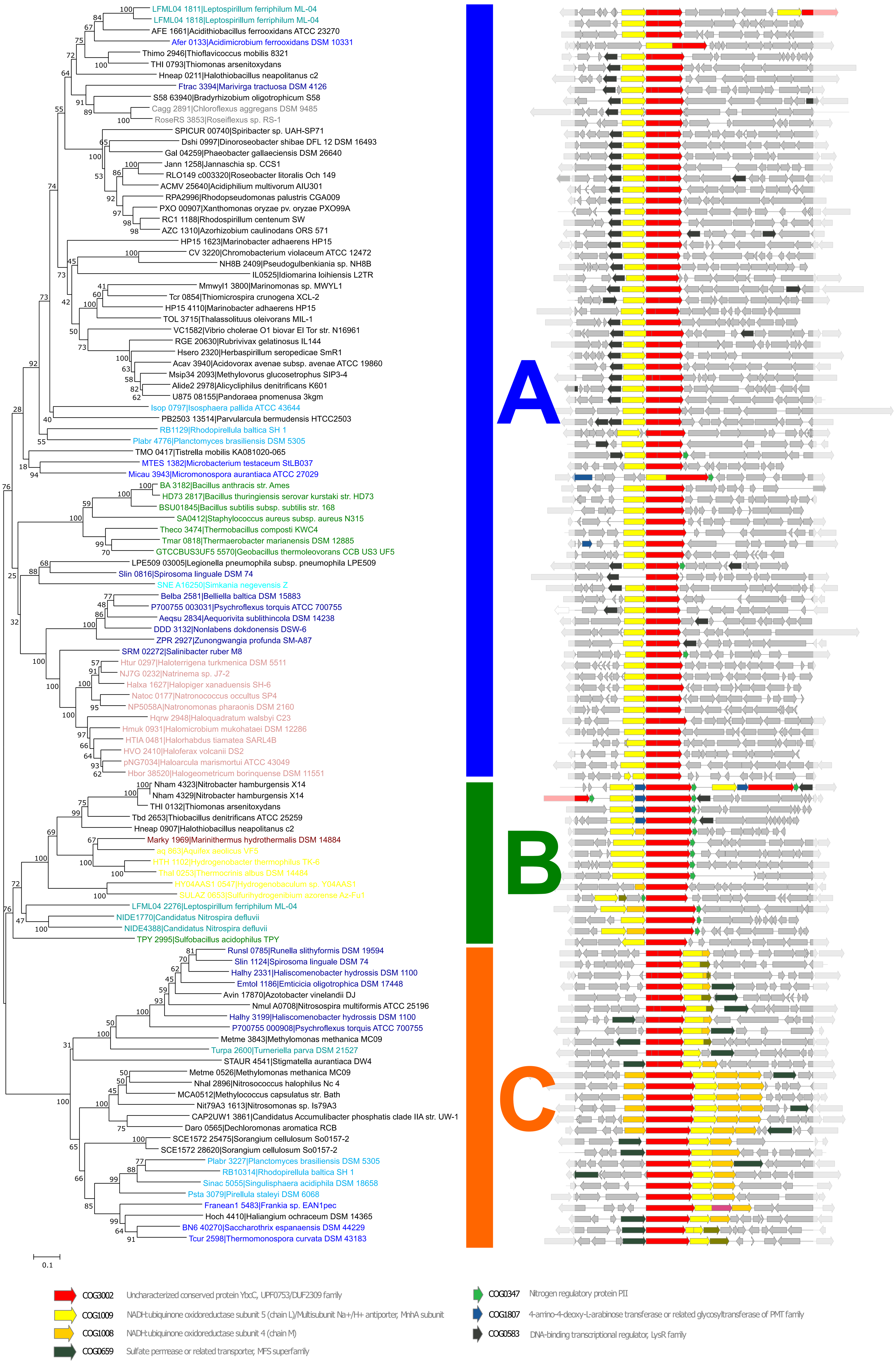

LFML04 1811|Leptospirillum ferriphilum ML-04
LFML04 1818|Leptospirillum ferriphilum ML-04
AFE 1661|Acidithiobacillus ferrooxidans ATCC 23270
Afer 0133|Acidimicrobium ferrooxidans DSM 10331
Thimo 2946|Thioflavicoccus mobilis 8321
THI 0793|Thiomonas arsenitoxydans
Hneap 0211|Halothiobacillus neapolitanus c2
Ftrac 3394|Marivirga tractuosa DSM 4126
S58 63940|Bradyrhizobium oligotrophicum S58
Cagg 2891|Chloroflexus aggregans DSM 9485
RoseRS 3853|Roseiflexus sp. RS-1
SPICUR 00740|Spiribacter sp. UAH-SP71
Dshi 0997|Dinoroseobacter shibae DFL 12 DSM 16493
Gal 04259|Phaeobacter gallaeciensis DSM 26640
Jann 1258|Jannaschia sp. CCS1
RLO149 c003320|Roseobacter litoralis Och 149
ACMV 25640|Acidiphilium multivorum AIU301
RPA2996|Rhodopseudomonas palustris CGA009
PXO 00907|Xanthomonas oryzae pv. oryzae PXO99A
RC1 1188|Rhodospirillum centenum SW
AZC 1310|Azorhizobium caulinodans ORS 571
HP15 1623|Marinobacter adhaerens HP15
CV 3220|Chromobacterium violaceum ATCC 12472
NH8B 2409|Pseudogulbenkiania sp. NH8B
IL0525|Idiomarina loihiensis L2TR
Mmwyl1 3800|Marinomonas sp. MWYL1
Tcr 0854|Thiomicrospira crunogena XCL-2
HP15 4110|Marinobacter adhaerens HP15
TOL 3715|Thalassolituus oleivorans MIL-1
VC1582|Vibrio cholerae O1 biovar El Tor str. N16961
RGE 20630|Rubrivivax gelatinosus IL144
Hsero 2320|Herbaspirillum seropedicae SmR1
Acav 3940|Acidovorax avenae subsp. avenae ATCC 19860
Msip34 2093|Methylovorus glucosetrophus SIP3-4
Alide2 2978|Alicycliphilus denitrificans K601
U875 08155|Pandoraea pnomenusa 3kgm
Isop 0797|Isosphaera pallida ATCC 43644
PB2503 13514|Parvularcula bermudensis HTCC2503
RB1129|Rhodopirellula baltica SH 1
Plabr 4776|Planctomyces brasiliensis DSM 5305
TMO 0417|Tistrella mobilis KA081020-065
MTES 1382|Microbacterium testaceum StLB037
Micau 3943|Micromonospora aurantiaca ATCC 27029
BA 3182|Bacillus anthracis str. Ames
HD73 2817|Bacillus thuringiensis serovar kurstaki str. HD73
BSU01845|Bacillus subtilis subsp. subtilis str. 168
SA0412|Staphylococcus aureus subsp. aureus N315
Theco 3474|Thermobacillus composti KWC4
Tmar 0818|Thermaerobacter marianensis DSM 12885
GTCCBUS3UF5 5570|Geobacillus thermoleovorans CCB US3 UF5
LPE509 03005|Legionella pneumophila subsp. pneumophila LPE509
Slin 0816|Spirosoma linguale DSM 74
SNE A16250|Simkania negevensis Z
Belba 2581|Belliella baltica DSM 15883
P700755 003031|Psychroflexus torquis ATCC 700755
Aeqsu 2834|Aequorivita sublithincola DSM 14238
DDD 3132|Nonlabens dokdonensis DSW-6
ZPR 2927|Zunongwangia profunda SM-A87
SRM 02272|Salinibacter ruber M8
Htur 0297|Haloterrigena turkmenica DSM 5511
NJ7G 0232|Natrinema sp. J7-2
Halxa 1627|Halopiger xanaduensis SH-6
Natoc 0177|Natronococcus occultus SP4
NP5058A|Natronomonas pharaonis DSM 2160
Hqrw 2948|Haloquadratum walsbyi C23
Hmuk 0931|Halomicrobium mukohataei DSM 12286
HTIA 0481|Halorhabdus tiamatea SARL4B
HVO 2410|Haloferax volcanii DS2
pNG7034|Haloarcula marismortui ATCC 43049
Hbor 38520|Halogeometricum borinquense DSM 11551
Nham 4323|Nitrobacter hamburgensis X14
Nham 4329|Nitrobacter hamburgensis X14
THI 0132|Thiomonas arsenitoxydans
Tbd 2653|Thiobacillus denitrificans ATCC 25259
Hneap 0907|Halothiobacillus neapolitanus c2
Marky 1969|Marinithermus hydrothermalis DSM 14884
aq 863|Aquifex aeolicus VF5
HTH 1102|Hydrogenobacter thermophilus TK-6
Thal 0253|Thermocrinis albus DSM 14484
HY04AAS1 0547|Hydrogenobaculum sp. Y04AAS1
SULAZ 0653|Sulfurihydrogenibium azorense Az-Fu1
LFML04 2276|Leptospirillum ferriphilum ML-04
NIDE1770|Candidatus Nitrospira defluvii
NIDE4388|Candidatus Nitrospira defluvii
TPY 2995|Sulfobacillus acidophilus TPY
Runsl 0785|Runella slithyformis DSM 19594
Slin 1124|Spirosoma linguale DSM 74
Halhy 2331|Haliscomenobacter hydrossis DSM 1100
Emtol 1186|Emticicia oligotrophica DSM 17448
Avin 17870|Azotobacter vinelandii DJ
Nmul A0708|Nitrosospira multiformis ATCC 25196
Halhy 3199|Haliscomenobacter hydrossis DSM 1100
P700755 000908|Psychroflexus torquis ATCC 700755
Metme 3843|Methylomonas methanica MC09
Turpa 2600|Turneriella parva DSM 21527
STAUR 4541|Stigmatella aurantiaca DW4
Metme 0526|Methylomonas methanica MC09
Nhal 2896|Nitrosococcus halophilus Nc 4
MCA0512|Methylococcus capsulatus str. Bath
Nit79A3 1613|Nitrosomonas sp. Is79A3
CAP2UW1 3861|Candidatus Accumulibacter phosphatis clade IIA str. UW-1
Daro 0565|Dechloromonas aromatica RCB
SCE1572 25475|Sorangium cellulosum So0157-2
SCE1572 28620|Sorangium cellulosum So0157-2
Plabr 3227|Planctomyces brasiliensis DSM 5305
RB10314|Rhodopirellula baltica SH 1
Sinac 5055|Singulisphaera acidiphila DSM 18658
Psta 3079|Pirellula staleyi DSM 6068
Franean1 5483|Frankia sp. EAN1pec
Hoch 4410|Haliangium ochraceum DSM 14365
BN6 40270|Saccharothrix espanaensis DSM 44229
Tcur 2598|Thermomonospora curvata DSM 43183
0.1
A
B
C
COG3002 Uncharacterized conserved protein YbcC, UPF0753/DUF2309 family
COG1009 NADH:ubiquinone oxidoreductase subunit 5 (chain L)/Multisubunit Na+/H+ antiporter, MnhA subunit
COG1008 NADH:ubiquinone oxidoreductase subunit 4 (chain M)
COG0659 Sulfate permease or related transporter, MFS superfamily
COG0347 Nitrogen regulatory protein PII
COG1807 4-amino-4-deoxy-L-arabinose transferase or related glycosyltransferase of PMT family
COG0583 DNA-binding transcriptional regulator, LysR family
