# Supplementary material for: COGNAT: a web server for comparative analysis of genomic neighborhoods
Source: Biol Direct. 2017 Nov 22;12:26. doi: 10.1186/s13062-017-0196-z (PMC5700660; doi:10.1186/s13062-017-0196-z)
Supplement: Supplementary file 2 — A part of a multiple sequence alignment of proteins belonging to the COG3002 (uncharacterized conserved protein YbcC, UPF0753/DUF2309 family). Only conserved blocks' regions with at least 95% conserved charged residues are shown (see the scheme below for their coordinates in the YbcC protein from Bacillus subtilis). Such conserved residues, which could be catalytically important, are marked with the “X” sign in the SITE pseudo-sequence. The multiple alignment was constructed with the MUSCLE software [5] and visualized with the help of the GeneDoc software (DOCX 225 kb) [file 13062_2017_196_MOESM2_ESM.docx]

Supplementary information to the paper: Klimchuk *et al.*, 2017

***Figure S2***. Part of the multiple sequence alignment of proteins belonging to the COG3002 (uncharacterized conserved protein YbcC, UPF0753/DUF2309 family). Only conserved blocks regions with at least 95% conserved charged residues are shown (see the scheme below for their coordinates in YbcC protein from *Bacillus subtilis*). Such conserved residues which could possibly by catalytically important are marked with the "X" sign in the SITE pseudo-sequence.

Multiple alignment was constructed with Muscle (<http://www.ebi.ac.uk/Tools/msa/muscle/>) and visualized in GeneDoc.


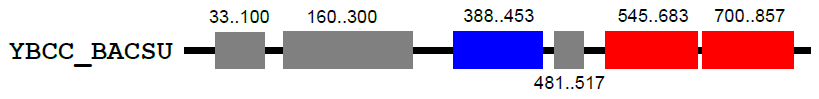


BLOCKS : ---BBBBBBBBBBBBBBBBBBBBBBBBBBBBBBBBBBBBBBBBBBBBBBBBBBBBBBBBBBBBBBBBBBBBBBBBBBBBBBBBBBBBBBBBBB------ :
SITE : ---------X-X-X----XX--X-------X-------------------------------------------------------------------- :
LFML04_1811|Leptospirillum_ferriphilum_ML-04 : QS-LVQAVFCIDVRSEIFRRALETVSPSIRTH----GFAGFFGVLVEFQPFGADSAKGHLPIL---------------------FNPSYRVEE------ :
LFML04_1818|Leptospirillum_ferriphilum_ML-04 : RP-DGQAVFCIDVRSETFRRALETVAPTVSTH----GFAGFFGVLVELLPFGGAVPKGHLPIL---------------------FNPTYRIRE------ :
AFE_1661|Acidithiobacillus_ferrooxidans_ATCC_23270 : RP-VAQAAFCIDVRSEIIRRALETVAPGIQTL----GFAGFFGVLMEYVPFGSNAPKGHLPVI---------------------FNPPYRVCE------ :
Afer_0133|Acidimicrobium_ferrooxidans_DSM_10331 : RP-RAQLAFCIDVRSEGIRRAVEARVAGAQTL----GFAGFFGVQMEYVPFGTEHARAHLPVI---------------------FAPPYRIRE------ :
Thimo_2946|Thioflavicoccus_mobilis_8321 : RP-AVQAAFCIDVRSEVFRRALETVAPGVQTV----GFAGFFGIFIEHVPLGSTAARSHVPVI---------------------FNPAYRVCD------ :
THI_0793|Thiomonas_arsenitoxydans : RP-AVQAAFCIDVRSEIFRRSLEIVAPSIQTI----GFAGFFGIFIEVVPLGADCGHSHVPIL---------------------FTPSYCLQE------ :
Hneap_0211|Halothiobacillus_neapolitanus_c2 : RP-TVQAAFCIDVRSEVFRRHLEASSPGLETI----GFAGFFGLPIDYCRMGESEARLQNPVL---------------------INPAYRAQE------ :
Ftrac_3394|Marivirga_tractuosa_DSM_4126 : KP-KVQAVFCIDVRSEVYRRNLESVAPEVGTL----GFAGFFGFPINFVKIGHDKGYDQCPAL---------------------IPSSYTVRE------ :
S58_63940|Bradyrhizobium_oligotrophicum_S58 : RP-PLQAAFCIDVRSEIFRRALETVYPHAETI----GFAGFFGFPIEYVPIGHASGGAQCPVL---------------------LKPAFIVCE------ :
Cagg_2891|Chloroflexus_aggregans_DSM_9485 : RK-RVQAAFCIDVRSEIFRRALETVSGEIETI----GFAGFFGFPIEYIPLAEVEGGAQCPVL---------------------LTPQFVITE------ :
RoseRS_3853|Roseiflexus_sp._RS-1 : RK-RVQAAFCIDVRSEIFRRALETVTDEIETI----GFAGFFGFPIEYVPLAETRGGAQCPVL---------------------LTPQFVIAE------ :
SPICUR_00740|Spiribacter_sp._UAH-SP71 : RP-ALQAAFCIDVRSEVFRRALEQVVPAAQTM----GFAGFFGVPVTHQGFASDLQESRLPAL---------------------LQPSLHSRA------ :
Dshi_0997|Dinoroseobacter_shibae_DFL_12__DSM_16493 : RP-ALQAAFCIDVRSEVIRRALETCDPGIETL----GFAGFFGLTAAHTPTGSCNSEARLPVL---------------------LTAGVTSKA------ :
Gal_04259|Phaeobacter_gallaeciensis_DSM_26640 : RP-AVQAAFCIDVRSEVFRRALEAQDSGVETI----GFAGFFGLATAHNAAGSDVIEGRGPVL---------------------LTAGVCSNA------ :
Jann_1258|Jannaschia_sp._CCS1 : RP-TLQAAFCIDVRSEVFRRALEAVNPGIQTL----GFAGFFGLTASHKSFASDVDELRLPVL---------------------LNAGVTSTS------ :
RLO149_c003320|Roseobacter_litoralis_Och_149 : RP-ALQAAFCIDVRSEVFRRALESLTPEIKTL----GFAGFFGLTPAHKGFASDVDELRLPVL---------------------LNPGLTSTS------ :
ACMV_25640|Acidiphilium_multivorum_AIU301 : RP-ALQTAFCIDVRSEVFRRALESMDPRIQTI----GFAGFFGLTTSHRRFASDVEERRLPVL---------------------LNPALRSYS------ :
RPA2996|Rhodopseudomonas_palustris_CGA009 : RP-ALQMAFCIDVRSEPFRRALESLDPRIRTL----GFGGFFGLPIAHRRFASDVVEARLPVL---------------------LPPRVTTSC------ :
PXO_00907|Xanthomonas_oryzae_pv._oryzae_PXO99A : RL-TLQMAFCIDVRSEVFRRALESLDSGITTL----GFAGFFGFGIGHRRFASDVVEARLPVL---------------------LSPGVVTCA------ :
RC1_1188|Rhodospirillum_centenum_SW : RP-ALQMAFCIDVRSEVFRRALESLDPGIRTL----GFAGFFGLGIGHRRFGSDVVEARLPVL---------------------LRPGVFTCS------ :
AZC_1310|Azorhizobium_caulinodans_ORS_571 : RP-TLQMAFCIDVRSEVFRRALESLDPGIRTL----GFAGFFGLGIGHRRFGSDVVEARLPVL---------------------LKPGIFTCS------ :
HP15_1623|Marinobacter_adhaerens_HP15 : IP-AAQAVFCIDVRSEVMRRHLEAVCPDVQTL----GFAGFFGMPIDHQQHGPLAPARRLPGL---------------------LPASFRLID------ :
CV_3220|Chromobacterium_violaceum_ATCC_12472 : RP-AMQAVFCIDVRSEPLRRALEQTVPASRTY----GFAGFFGLPLAYRVPGSDVAQPRLPVL---------------------LAPGWEASA------ :
NH8B_2409|Pseudogulbenkiania_sp._NH8B : GR-ELHAVFCIDVRSEPMRRALEQEIPEASSA----GFAGFFGLPLAIRFAGETRTQPRLPGL---------------------LAPSWEALA------ :
IL0525|Idiomarina_loihiensis_L2TR : AP-DVQAVFCIDVRSERYRRALEQAGKSLGSYVQSKGFAGFFGVPLAIQRKGRDV--PHVPGL---------------------LQPAYYIKA------ :
Mmwyl1_3800|Marinomonas_sp._MWYL1 : RP-ELQAIFCIDVRSEVFRRALEQQSATIQTL----GFAGFFGLPIEYKAKDSHYVRPQLPGL---------------------LQAAITVTE------ :
Tcr_0854|Thiomicrospira_crunogena_XCL-2 : -PVLLQAAFCIDVRSEVIRRALEAQDSRVETL----GFAGFFGLPIEYQPAGTDVSRPQLPGL---------------------LKSGIKVTP------ :
HP15_4110|Marinobacter_adhaerens_HP15 : QPLKLQAAFCIDVRSEVYRRALEAQNPAIQTL----GFAGFFGLPISYRPKGTGFCRPQLPGL---------------------LAPALEVTE------ :
TOL_3715|Thalassolituus_oleivorans_MIL-1 : QP-KLQAVFCIDVRSEVMRRAFEAQSSKIQTF----GFAGFFGIPLVYTPVGTDLNRPQLPGL---------------------LAAGLRASE------ :
VC1582|Vibrio_cholerae_O1_biovar_El_Tor_str._N1696 : YP-EVQAAFCIDVRSEVIRRHLEAQSPHIQTL----GFAGFFGLPIRYQLLGTEASRPQLPGL---------------------LAPSLIVSD------ :
RGE_20630|Rubrivivax_gelatinosus_IL144 : AP-EAQAVFCIDVRSEPLRRALEAASPGVQTF----GFAGFFGLPLAYTPLATGARRPQLPGL---------------------LAPGVEAVE------ :
Hsero_2320|Herbaspirillum_seropedicae_SmR1 : AI-EVQAAFCIDVRSEPMRRALERLHPGVQTL----GVAGFFGLPVAYTPLGTQAARPQLPGL---------------------LAPAYQVQE------ :
Acav_3940|Acidovorax_avenae_subsp._avenae_ATCC_198 : EP-EAQAVFCIDVRSEPLRRALEATAPTLQTL----GFAGFFGVPAAYTPLGTTARRPQLPGL---------------------LPPAMEVTD------ :
Msip34_2093|Methylovorus_glucosetrophus_SIP3-4 : AI-EVQAAFCIDVRSEPLRRALETAWPGIQTL----GFAGFFGLPVAYTPLATQARRPQLPGL---------------------LAPAMEVVD------ :
Alide2_2978|Alicycliphilus_denitrificans_K601 : DI-EVQAAFCIDVRSEPMRRALEAVWPGIQTL----GFAGFFGLPVAYTPLASQARRPQLPGL---------------------LAPAIEVTD------ :
U875_08155|Pandoraea_pnomenusa_3kgm : DI-EVQAAFCIDVRSEPLRRSLEAVSPGIQTL----GFAGFFGLPIAYTPLGTPARRPQLPGL---------------------LAPAIEVTD------ :
Isop_0797|Isosphaera_pallida_ATCC_43644 : RP-RVQAVFCIDVRSEPLRRHLEAVASDIETR----GFAGFFGVALAWRSEGKT--DARCPVL---------------------LKPGVTVEH------ :
PB2503_13514|Parvularcula_bermudensis_HTCC2503 : RP-SIQAVFCIDVRSERLRHAVEAEDPLAQTY----GFAGFFGVPLSVLTKTAER-RDHCPVL---------------------LSPTLHCTH------ :
RB1129|Rhodopirellula_baltica_SH_1 : KP-AVQMAFCIDVRSERFRRHLEQVDASVDTL----GIAGFFGLPFEYVPLGQSSGDTHAPVL---------------------LSPKFALRE------ :
Plabr_4776|Planctomyces_brasiliensis_DSM_5305 : RK-RAQLVFCIDVRSELLRRNLEATSEGIETF----GFAGFFGLPISHVPFSQKHGHAHCPVL---------------------IEPSLIAQD------ :
TMO_0417|Tistrella_mobilis_KA081020-065 : QP-LGQIVLCIDVRSEVLRRHLEATGP-WATL----GFAGFFGVPVAVRPFGEDDPHASCPVL---------------------LRPRHLVDE------ :
MTES_1382|Microbacterium_testaceum_StLB037 : RP-LAQAVFCIDTRSESFRRHLEAAGP-VETL----GFAGFFAVPISFRPADGSGEIASCPVL---------------------LTPRVAITE------ :
Micau_3943|Micromonospora_aurantiaca_ATCC_27029 : TP-LAQAVFCIDVRSEGLRRHLEAAGP-VDTY----GFAGFFGLPVRTVAADAARGRDRCPVL---------------------MRPVATVGE------ :
BA_3182|Bacillus_anthracis_str._Ames : RV-VAQLAFCIDVRSEPFRRHLEKLGP-FETF----GIAGFFGLPIATTELGSNDSHPSLPVI---------------------LKPKHQIKE------ :
HD73_2817|Bacillus_thuringiensis_serovar_kurstaki_ : RV-LAQLAFCIDVRSEPFRRHLEKLGP-FETF----GIAGFFGLPIATSELGSNDSHPSLPVI---------------------LKPKHQIKE------ :
BSU01845|Bacillus_subtilis_subsp._subtilis_str._16 : SA-LAQLAFCIDVRSEPFRRQLEKEGP-FETI----GIAGFFGVPIATCELGSKHSHASLPII---------------------QKPQNKIKE------ :
SA0412|Staphylococcus_aureus_subsp._aureus_N315 : ST-KAQIAFCIDVRSEPFRRHIEAAGP-FETI----GIAGFFGLPIQKDAVDEQFKHDSLPVM---------------------VPPAYRIKE------ :
Theco_3474|Thermobacillus_composti_KWC4 : RA-AAQLLFCIDVRSEPFRRHLEREGP-FETF----GCAGFFGLPIRTRLPDG-HVHAACPAI---------------------VEPRHEVRE------ :
Tmar_0818|Thermaerobacter_marianensis_DSM_12885 : AP-AVQLLFCIDVRSEPLRRHLERAGP-FETY----GCAGFFNLPIRKRELDSPYAHPSCPAI---------------------VEPQHEIAE------ :
GTCCBUS3UF5_5570|Geobacillus_thermoleovorans_CCB_U : QA-AAQLLFCIDVRSEPFRRHVEAVGP-FETY----GCAGFFGLPIQTRVLDSDDAHPSCPAI---------------------VAPRHEINE------ :
LPE509_03005|Legionella_pneumophila_subsp._pneumop : RA-KAQMVFCIDVRSEPFRRCIEKLGH-YETL----GFAGFFGLPVSIKDYDGETIKDSCPVL---------------------LKPRFNIHE------ :
Slin_0816|Spirosoma_linguale_DSM_74 : TP-DAQLVFCIDVRSEPFRRALEATGD-YQTL----GFAGFFGVPVQITDTVTGETHASCPVL---------------------LSPKHTVHE------ :
SNE_A16250|Simkania_negevensis_Z : QT-KAQFIFCIDVRSEPIRREIESIGG-YETF----GAAGFFGLPIAVKPYGSDAFLTACPAI---------------------VKPQYKVQE------ :
Belba_2581|Belliella_baltica_DSM_15883 : SP-EAQFVFCIDTRSELIRRHVEACGN-YETF----GYAGFFGIAMDYENLSDGIVKKSCPPI---------------------LASSYKVSE------ :
P700755_003031|Psychroflexus_torquis_ATCC_700755 : -P-EAQLVFCIDTRSELIRRNVESKGH-YETF----GYAGFFGIPMNYKPLNNEIIRKSCPPI---------------------LDSAYSVSE------ :
Aeqsu_2834|Aequorivita_sublithincola_DSM_14238 : -P-DAQFALCIDTRSESMRRHIENVGN-YETF----GYAGFFGIAMDYKNENDGLVRKSCPPI---------------------LGSAYIVSE------ :
DDD_3132|Nonlabens_dokdonensis_DSW-6 : -P-DAQLVFCIDTRSELIRRHVEAQGN-YETY----GYAGFFGIAMDYTNLEDNITRKSCPPI---------------------VGSAYEVTE------ :
ZPR_2927|Zunongwangia_profunda_SM-A87 : -P-DAQMVFCIDTRSELIRRKIEETDN-YETF----GYAGFFGIAMDYKSTEDGLTRKSCPPI---------------------LNSAYKVSY------ :
SRM_02272|Salinibacter_ruber_M8 : RP-DAQLVFCIDTRSEVIRRHIEQQGP-YETH----GYAGFFGVPMQHQPYGTEERVKSCPPI---------------------VDPKHRIME------ :
Htur_0297|Haloterrigena_turkmenica_DSM_5511 : RP-AAQLVFCIDTRSEVIRRHIEAQGP-YETH----GYAGFFGVPMRHRKHDSHAETDACPPI---------------------VDPQHRVVD------ :
NJ7G_0232|Natrinema_sp._J7-2 : RP-AAQLVFCIDTRSEVIRRHIEVQGP-YETH----GYAGFFGVPMRHRGYEAAADTDACPPI---------------------VEPEHRIVD------ :
Halxa_1627|Halopiger_xanaduensis_SH-6 : RP-AAQLVFCIDTRSEIIRRHIEDQGS-YETH----GYAGFFGVPMRYRGYDAKADVDACPPI---------------------VDAEHRVVD------ :
Natoc_0177|Natronococcus_occultus_SP4 : RP-DAQLVFCIDTRSEIIRRHIEAQGS-YETH----GYAGFFGIPMQYKEHDSEVVADACPPI---------------------VEPQHLIAD------ :
NP5058A|Natronomonas_pharaonis_DSM_2160 : RP-AAQLVFCIDTRSEIIRRHIEQQGP-YETH----GYAGFFGVPMRHEAYGSDVTTEACPPI---------------------VEPEHIIAD------ :
Hqrw_2948|Haloquadratum_walsbyi_C23 : RP-DAQLVFCIDTRSEIIRRHIESTGQ-YETY----GYAGFFGIPMRYRGYDDAVSIDACPPI---------------------VDAQHRISE-----S :
Hmuk_0931|Halomicrobium_mukohataei_DSM_12286 : RP-DAQLVFCIDTRSEIIRRHVEAAGD-YETH----GYAGFFGVPMRYEGHDSEVAVDACPPI---------------------LDPQHRIAD------ :
HTIA_0481|Halorhabdus_tiamatea_SARL4B : RP-DAQLVFCIDTRSEVIRRHVEATGD-YETH----GYAGFFGVPMEYQAYDADVSVDAAPPI---------------------VDPAHRITE------ :
HVO_2410|Haloferax_volcanii_DS2 : RP-DAQLVFCIDTRSEVIRRHIEATGD-YETH----GYAGFFGIPMEYRGYDAEVAVDACPPI---------------------VDPQHRVTE------ :
pNG7034|Haloarcula_marismortui_ATCC_43049 : RP-DAQMVFCIDTRSEIIRRHIEATGD-YETH----GYAGFFGIPMEYQGYDTDVSVDACPPI---------------------LDPQHHVTD------ :
Hbor_38520|Halogeometricum_borinquense_DSM_11551 : RP-DAQLVFCIDTRSEVIRRHIEATGD-YETH----GYAGFFGIPMEYQGYDDDVSVDVCPPI---------------------LDPQHHIID------ :
Nham_4323|Nitrobacter_hamburgensis_X14 : RP-FAQAWFCIDVRAEPIRRHLERVGD-YQTF----GIAGFFGVPVGFLGYGKGSESHFCPAV---------------------VTPKNLVLE------ :
Nham_4329|Nitrobacter_hamburgensis_X14 : RP-FAQAWFCIDVRAEPIRRHLERVGD-YQTF----GIAGFFGVPVGFLGYGKGSESHFCPAV---------------------VTPKNLVLE------ :
THI_0132|Thiomonas_arsenitoxydans : RP-FAQAWFCIDVRSEPIRRHLERVGD-YQTF----GIAGFFGVPVGFLGYGKGSESHFCPAV---------------------VTPKNLVLE------ :
Tbd_2653|Thiobacillus_denitrificans_ATCC_25259 : RP-FAQVMFCIDVRSERIRRHLEKLGS-YQTF----GIAGFFGVPVSFIGLEKGSETHLCPVV---------------------ATPKNVVLE------ :
Hneap_0907|Halothiobacillus_neapolitanus_c2 : RP-FAQALFCIDVRSEPIRRNLETVGE-YQTY----GIAGFFGVPVSYIGLGKGSEVNLCPVV---------------------ITPKNLVLE------ :
Marky_1969|Marinithermus_hydrothermalis_DSM_14884 : SP-KAQVLFCIDVRSERYRRNLERLGR-YETF----GVAGFFGVPMAFVELHKGHEEFLCPAL---------------------IRPRNVVLD------ :
aq_863|Aquifex_aeolicus_VF5 : KP-KAQALFCIDVRSERYRRNLEKIGN-YETY----GIAGFFGVPMAFVEIHKGHEEFLCPVL---------------------IKPRNVVLE------ :
HTH_1102|Hydrogenobacter_thermophilus_TK-6 : KP-LAQALFCIDVRSERFRRKLESVGN-YQTF----GVAGFFGIPVAMVDLRKGHEEFLCPVI---------------------VTPKNVVFE------ :
Thal_0253|Thermocrinis_albus_DSM_14484 : RP-LAQAFFCIDVRSERFRRHLESLGR-YQTY----GIAGFFGVPVAMVNLQKGHEEFLCPVI---------------------VTPRNVVFE------ :
HY04AAS1_0547|Hydrogenobaculum_sp._Y04AAS1 : DI-LASAVFCIDVRSEAIRRHIERLGN-YNTY----GVAGFFGTPIAFIEFDKGHEQYLCPAL---------------------IKPQKIIFE------ :
SULAZ_0653|Sulfurihydrogenibium_azorense_Az-Fu1 : KP-KAFAVFCVDVRSEALRRNLERVDN-YKTF----GVAGFFGVKMALIEFDKAHELLLCPAM---------------------EIPDKVVLE------ :
LFML04_2276|Leptospirillum_ferriphilum_ML-04 : PPTRAQLLFCIDVRSEGIRRHLERSGG-YETA----GLAGFFGIPIRFKGFGSDHVQVLSPAI---------------------LSPRHFLAE------ :
NIDE1770|Candidatus_Nitrospira_defluvii : RP-LAQMVFCIDVRSEVLRRHLEQLGG-YETL----GVAGFFGIPVKYQAFGEEHPVTHSPVL---------------------LKPKNHIRE------ :
NIDE4388|Candidatus_Nitrospira_defluvii : RP-HSQSVFCIDVRSEPFRRHLESTGA-NDTY----GFAGFFAVFIRYRAWSKEHETEQFPVI---------------------MRAKNEVRE------ :
TPY_2995|Sulfobacillus_acidophilus_TPY : PA-PTQVAFCMDVRSEGIRRHLEAQGA-YETI----GAAGFFGIPMTYQEWMGHRPTHRYPAI---------------------LTPQMAVRE------ :
Runsl_0785|Runella_slithyformis_DSM_19594 : ---SFQAMFCIDDRECSLRRYLEDLDPACETF----GTPGFFGVEFFYQPEDGKFYTKVCPAP---------------------VTPKYLIKE------ :
Slin_1124|Spirosoma_linguale_DSM_74 : ---TFQAMFCIDDRECSFRRYLEDLDHSCETF----GTPGFFGVDCFFQPEEGKYYTKICPVP---------------------LSPKHLIKE------ :
Halhy_2331|Haliscomenobacter_hydrossis_DSM_1100 : ---NFQAAFCIDDREGSLRRYLELFDPECETF----GTPGFFGVEFYFQPQGGKFFTKTCPAP---------------------MHPKFLIRE------ :
Emtol_1186|Emticicia_oligotrophica_DSM_17448 : QS-SFQAVFCIDDRECSIRRYVEHLDEHCRTY----GTAGFFGVEFFYKPVHANSSTKQCPAP---------------------VTPQYLIKE------ :
Avin_17870|Azotobacter_vinelandii_DJ : ---SFQGLFCIDDRICSFRRHIESLDPHCETY----GTPGFFGVEFYFKPENAKSHTKVCPGS---------------------IEPRYLIKE------ :
Nmul_A0708|Nitrosospira_multiformis_ATCC_25196 : ---SFQTYFCIDDRLTSFRRYLEQLDPDCETF----TTAGFFNVELYYQPENGKFYTKCCPAP---------------------VFPKFLVKE------ :
Halhy_3199|Haliscomenobacter_hydrossis_DSM_1100 : ---SFQAVFCIDERECSLRRHIESIDPHCETL----GAPGFFGVEFFFQPENGQFYEKLCPAP---------------------VTPAYLIKE------ :
P700755_000908|Psychroflexus_torquis_ATCC_700755 : ---SFQAFFCIDDREESIRRHLEQASPDCETF----GTPGHFGMVAMYQPESGKFYTQVCPGS---------------------LTPKHLIKE------ :
Metme_3843|Methylomonas_methanica_MC09 : ---EVQAIFCIDDRECSLRRYLEEVNPHIETF----GAPGFFGIDFLYQGLDDVYPVAQCPVV---------------------IKPRHLITE------ :
Turpa_2600|Turneriella_parva_DSM_21527 : TA-DFQAIFCIDDREESIRRHLEEVNQKIETF----GAPGFFGIDMVYQGPFDAIAIKQCPVP---------------------VTPKYRVRG------ :
STAUR_4541|Stigmatella_aurantiaca_DW4 : AP-RFQALMCMDDREESMRRALESKECGIETW----GYVGFFNVDMRFEAVGASRATRQCPPV---------------------VEPSRTIQE------ :
Metme_0526|Methylomonas_methanica_MC09 : AP-QAQIVFCMDDREEGIRRHLEELNPAVETL----GAAGFFGVPMHYQGLDDAHATPLCPVV---------------------VTPSHTVRE------ :
Nhal_2896|Nitrosococcus_halophilus_Nc_4 : RP-EAQMIFCIDDREESIRRHLEEINPNIETL----AAAGFFGVKINWRGLDDTKLTPLCPVVFKSARELNENPHAERPISIDWVTPPHEVRE------ :
MCA0512|Methylococcus_capsulatus_str._Bath : RP-QAQIVFCMDDREEGVRRHLEELNPAVETL----GAAGFFGIPMYWQGLDDAEPSALCPIV---------------------VIPSHEVRE------ :
Nit79A3_1613|Nitrosomonas_sp._Is79A3 : RP-QAQIVTCMDDREESLRRQLEEINPAIETI----GAAGFFGIPMNYKGIDDTEIKMQCPVV---------------------VRPANDVNE------ :
CAP2UW1_3861|Candidatus_Accumulibacter_phosphatis_ : GA-QAQFVLCMDDREEGTRRHLEEVNPAFETF----GAAGFFGVPMLWQGLDDEVPTALCPIV---------------------VRPENAVRE------ :
Daro_0565|Dechloromonas_aromatica_RCB : AP-SAQVVMCMDDREEGTRRHLEEIAPDVATY----GAAGFFGVPMFWQGLDDAGKTALCPVV---------------------VQPTHLLRE------ :
SCE1572_25475|Sorangium_cellulosum_So0157-2 : DP-RFQLAMCIDDRCEGLRRHFEELSPRHETL----GIAGFFGVPIRFRGLDDAGHVSLCPVG---------------------VEPAHEIVE------ :
SCE1572_28620|Sorangium_cellulosum_So0157-2 : EP-RFQVAFCIDDRCEGLRRHFEELSPRHETL----GVAGFFGVPIRYRGLDDAGHASLCPVG---------------------VEPAHEIVE------ :
Plabr_3227|Planctomyces_brasiliensis_DSM_5305 : RP-TFQITTCIDDREESFRRHLEEVQPHCETY----GAAGFFAVAMNYRGAAESFYKPLCPAV---------------------VTPQHFVKE------ :
RB10314|Rhodopirellula_baltica_SH_1 : RP-AYAAIFCIDDREESFRRHLEEVAPDCRTA----SAAGFFAVAMYYQGADHASFRPLCPAI---------------------VKPQHYVRE------ :
Sinac_5055|Singulisphaera_acidiphila_DSM_18658 : SP-AFQVACCLDEREESFRRHLEELAPSVETF----GAAGFYGVAMYYRGAADAHFVPLCPVV---------------------IRPQHWVTE------ :
Psta_3079|Pirellula_staleyi_DSM_6068 : KP-RFQVVCCIDAREESFRRHLEEVAPDVETF----GAPGFFGVAIYYRGLSDANFAALCPIV---------------------VRPKHWVIE------ :
Franean1_5483|Frankia_sp._EAN1pec : PP-DFQAVFCMDEREESLRRHLEESHPQVRTY----GASGYFGVAMAYQGLDDVRPRALCPVT---------------------MTPRSLVVE------ :
Hoch_4410|Haliangium_ochraceum_DSM_14365 : KA-RVQAAFCIDDREESLRRHFEEVMPDVETI----GFAGFYGAAMAYKGIEHVKPEPLCPVN---------------------IVPDRLVVE------ :
BN6_40270|Saccharothrix_espanaensis_DSM_44229 : TP-AFQAVFCLDEREESVRRHLEECCPAVETF----GFAGFFGVAMNYRGVADVRPRPLCPVV---------------------VTPRHAVVE------ :
Tcur_2598|Thermomonospora_curvata_DSM_43183 : PP-RFQAVFCIDEREEALRRHLEEHFLHVETF----GYAGSFGVAMLYRGMEDVRARPLCPAA---------------------VTPRHLVEE------ :

BLOCKS : ----BBBBBBBBBBBBBBBBBBBBBBBBBBBBBBBBBBBBBBBBBBBBBBBBBBBBBBBBBBBBBBBBBBBBBBBBBBBBBBBBBBBBBBBBBBBBBBB :
SITE : -----------------------------------X-----XX-------XXXX----------------X--------------------------X- :
LFML04_1811|Leptospirillum_ferriphilum_ML-04 : ----ADRPAVAEFALRNMGLT---GNFARLVLLVGHGSTTVNNPQATALDCGACAGQTGEASARIAAFLLNDPVTRRGLAQK-GIVIPEETWFVAGLHD :
LFML04_1818|Leptospirillum_ferriphilum_ML-04 : ----SDRPAVAESALRNMGLT---GNFARLVLLVGHGSTTTNNPQATALDCGACAGQTGEASARIAAMLLNDPVARRGLAQK-GVVLPEDTWFVAGLHN :
AFE_1661|Acidithiobacillus_ferrooxidans_ATCC_23270 : ----TERAGVGEFILKNMGLT---QTFARLILLAGHGSTTVNNPQGTGLDCGACAGQTGEASARIAVTLLNDPATRRGLEEK-GLKIPKDTYFIAGLHD :
Afer_0133|Acidimicrobium_ferrooxidans_DSM_10331 : ----DRLAEIGRFILTSMGLT---GGFAPLVVLVGHGSETVNNPQGSGLDCGACGGQTGEVSAKVAAALLNDPRTRAGLERV-GISVPSDTVFVAALHS :
Thimo_2946|Thioflavicoccus_mobilis_8321 : ----ADRVEHAERILRAMSMT---DGFARLVLLAGHGSTSVNNPHATGLDCGACAGQTGEANARVVATLFNDPTVRRGLRQR-GIAIPEDTWFLAGLHD :
THI_0793|Thiomonas_arsenitoxydans : ----DQRVENAERILRAMGLT---GPFARLVLLVGHGSSSVNNPHATSLDCGACAGQTGEASAKVVAALFNDKQVRVELARR-GILIPEDTWFLAALHD :
Hneap_0211|Halothiobacillus_neapolitanus_c2 : ----QEKVDLAEKVLRGLGLT---HTFAPIVLLAGHGSSTTNNPHRAGLDCGACAGQAGDVNARVAVQLLNEAAVRLGLIER-GIAIPRDTRFVAALHD :
Ftrac_3394|Marivirga_tractuosa_DSM_4126 : ----EARVNMAKGALQAMSLT---EDFARVVMIVGHGSTSVNNPHASGLDCGACAGQSGEANAKVASIILNDKEVRKQLASD-KIVIPDSTYFLACLHD :
S58_63940|Bradyrhizobium_oligotrophicum_S58 : ----AQRVAMAEAVLRAMSLT---GPFARLVLLAGHGSTTVNNPHASGLDCGACGGHTGEANARVAAAVLNDAGVREGLRAK-GIDVPADCWFIGALHD :
Cagg_2891|Chloroflexus_aggregans_DSM_9485 : ----PQRIAAAAGALKAMSLT---DNFARIVLLAGHGSTTVNNPHATGLDCGACGGHTGEANVRVAVQILNDPAVRAGLREH-GIVIPSDTVFVAGLHD :
RoseRS_3853|Roseiflexus_sp._RS-1 : ----EQRVAAAEGALKAMSLT---NNFARLVLLTGHGSTTVNNPHATGLDCGACGGHTGEANVRVAVRILNDPAVRAGLKER-GLIIPDDTVFLAGLHD :
SPICUR_00740|Spiribacter_sp._UAH-SP71 : ----RERVDTAETILRAMSLT---DGFAPLVLLMGHGASVTNNPHGSALQCGACGGYAGDVNARLLAGLLNDTSVRAGLKNR-GITFPEDTCFMAGLHD :
Dshi_0997|Dinoroseobacter_shibae_DFL_12__DSM_16493 : ----EAQIDAAATILNAMSLK---SNFAPLVVIAGHGSHVNNNAHASALQCGACGGYGGDVNARLLADLLNQPHVRAGLAAR-GIAVPEDTIFVAALHD :
Gal_04259|Phaeobacter_gallaeciensis_DSM_26640 : ----DSRIAMAKTILSAMSLT---DNFAPVVLLAGHGADVTNNPHASALHCGACGGHAGDVNARLLAQLLNDTGVRAGLTAV-GIVIPQDTVFLAALHH :
Jann_1258|Jannaschia_sp._CCS1 : ----DAQIDAAETILRAMSLT---TDFAPLVVLAGHGANVVNNPFASGLHCGACGGYAGDVNARLLAALLNTPDVRAGLADR-GIDVPSDTLFLGALHD :
RLO149_c003320|Roseobacter_litoralis_Och_149 : ----AAQTDAAETILRAMSFT---DNFARLVVLAGHGANVVNNPFASGLHCGACGGYSGEVNARLLAGLLNNVDVRSGLVER-GITIPEDTIFVGALHD :
ACMV_25640|Acidiphilium_multivorum_AIU301 : ----ATRVETAETVLRAMSLT---RDFARLVLFVGHGANVVNNPHASALHCGACGGYSGEVNARLLASLLNDAKVRGGLSRN-GIEIPDDTHFVAALHD :
RPA2996|Rhodopseudomonas_palustris_CGA009 : ----GARVDTAEAVLRAMSLT---GPFAPLVLIAGHGASVVNNPHASALHCGACGGFPGDVNARLLAGLLNDPQVRTALIGR-DIAIPADTLFVGALHD :
PXO_00907|Xanthomonas_oryzae_pv._oryzae_PXO99A : ----DTRLTMAARILKAMSFT---SNFARLVVLAGHGAKVVNNPHASALHCGACGGYSGEVNARLLASLLNDHQVRAGLAER-GIVIPADTLFLAALHD :
RC1_1188|Rhodospirillum_centenum_SW : ----DTRLGMAANVLRAMSLT---EGFARLVLLAGHGASVVNNPHASALHCGACGGYSGEVNARLLAALLNDRDVRAGLASQ-GIRIPDDTVFLGALHD :
AZC_1310|Azorhizobium_caulinodans_ORS_571 : ----DTRLTMAVSILKAMSLT---RGFARLVLLAGHGANVVNNPHASALHCGACGGYSGEVNARLLASVLNDREVRADLAER-GIIVPEDTLFLAALHD :
HP15_1623|Marinobacter_adhaerens_HP15 : ----AERVALAANMLRGMSLT---SGFAPLLVLVGHGSHTDNNPNQAGLDCGACGGQSGGVNARLAASLVNDPQVRAGLADE-GIRIPDFTWAVAAEHC :
CV_3220|Chromobacterium_violaceum_ATCC_12472 : ----RA--EIVSGLLPAMGLA---GALAPWVLLVGHASHVSNNPQAAALQCGACGGHGGHQHVRLLAGWLNDPALRERLAAL-GRAIPADTVFLPALHL :
NH8B_2409|Pseudogulbenkiania_sp._NH8B : ----GTIISVLTTLLPAMGLG---ADFPRTVLLIGHASHSSNNPQASALQCGACGGHGGHLHVLLLSDWLQRPAVREGLAAA-GLAIPSDTVFLPVLHL :
IL0525|Idiomarina_loihiensis_L2TR : ----ESLVGICQQALAGMQFT---R-FARHIVLVGHGSHHSNNAQRAGLNCGACGGQTGALSARVLVRLLNNQNIREHLREQ-GVAIPDATQFHSAMHE :
Mmwyl1_3800|Marinomonas_sp._MWYL1 : ----QDKAHLAKGILDTIKLT---T-YAPIVMLVGHGSHTSNNLHAAGLECGACGGQSGEVNVRVLASLLNDHKVRTLLNDM-GMEIPSDTQFVPALHN :
Tcr_0854|Thiomicrospira_crunogena_XCL-2 : ----DQKVELAAGILHAMGLD---HDLAETVMLVGHGSTSCNNPHAAGLDCGACGGQTGEINVRVLAFLLNDESVRQGLLEK-DIKIPAQTRFVAAMHN :
HP15_4110|Marinobacter_adhaerens_HP15 : ----AEKAELAGGILRAMTLT---HDFAPTVLLVGHGSSTRNNPHAAGLDCGACGGQTGSVNVRVLAGILNDKDVRAALAKQ-GISIPSETRFVGALHN :
TOL_3715|Thalassolituus_oleivorans_MIL-1 : ----VEKTDLVEGILKAMSIK---R-FASEVLLVGHGSHTTNNPHAAGLDCGACGGQTGAVNVQVLAQLLNNQGVRAALADR-GWVIPTSTRFIAALHN :
VC1582|Vibrio_cholerae_O1_biovar_El_Tor_str._N1696 : ----AQRAQMAANILRALGIA---TEQARLVLLVGHGSQTQNNPQRAGLDCGACCGQSGEVNARTLAALLNDQAVRQALPEY-GISLRDDVHFIAALHN :
RGE_20630|Rubrivivax_gelatinosus_IL144 : ----DEKVELAARVLRAMGLQ---RGLAPLVVFVGHGSQSANNAQAAALDCGACCGQSGEVNARVLARLLNEPAVREGLRGH-GIDVPARTVFVAALHN :
Hsero_2320|Herbaspirillum_seropedicae_SmR1 : ----ADKVALAARILHAMGLD---RAVAPLVLLVGHGSQSANNAHAAALDCGACCGQTGEVNARTLAQLLNEHSVRRGLLAQ-GVDLPEETAFLALLHN :
Acav_3940|Acidovorax_avenae_subsp._avenae_ATCC_198 : ----QERAALAARVLKGLGLA---RGAAPLVMLVGHGSQSANNAQAAALDCGACCGQTGEVNARALALLLNDAGVRAALPGL-GIALPEATRFVAALHN :
Msip34_2093|Methylovorus_glucosetrophus_SIP3-4 : ----EAKIDLAARVLQGMGLT---RELAPMVLLVGHGSQSANNAHAAGLDCGACCGQTGEVNARSLALLLNEPEVRQGLQAK-GIAVPSHTVFVAALHN :
Alide2_2978|Alicycliphilus_denitrificans_K601 : ----DAKVALAARVLHAMGLE---QHLAPLVLLVGHGSQSANNAHAAALDCGACCGQTGEVNARSLALLLNDPAVRQGLRGA-GVAIPDSTTFMACLHN :
U875_08155|Pandoraea_pnomenusa_3kgm : ----ASKVALATRVLHAMGLD---RYLAPLVLLVGHGSQSANNAHAAALDCGACCGQTGEVNARSLAQLLNDPAVRDGLRAQ-QLAIPEATRFVAVLHN :
Isop_0797|Isosphaera_pallida_ATCC_43644 : ----QAQLDLAVGILQNMGFA---DRFARLILLCGHESHSANNPHAAGLDCGACGGHGGAINARVAAALLNDPAIRTGLVQR-GWSLPSDTHFLPGVHD :
PB2503_13514|Parvularcula_bermudensis_HTCC2503 : ----DERTDLALRILKGMSLG---DNLARLVLLVGHDATTENNPYAAGLACGACGGHSGAPNAVIAARILNDPEVRTRLADR-GVALPSDTRFLAALHD :
RB1129|Rhodopirellula_baltica_SH_1 : ----DQQTDLVEGLLNSMGLS---DDFAPLVVLCGHGSQTDNNAMAAGLDCGACGGHSGAPNARLAAILLNDRRIQKRLSDR-GIEIPAETHVIAAWHN :
Plabr_4776|Planctomyces_brasiliensis_DSM_5305 : ----SEKADLAENILRNLGLT---ENFARLVVFCGHEAETTNNPYQSGLDCGACGGHSGGPNARAAANLLNDPDVRWELRSR-GLVIPVDTRFVAGVHN :
TMO_0417|Tistrella_mobilis_KA081020-065 : GLSPAEQAFFAEGALRVMGLT---KGFAELVVFCGHGGHTVNNVFASGLDCGACGGNRGGPNARILAALLNAPAVRAALAER-GIVIPEITRFLSAEHD :
MTES_1382|Microbacterium_testaceum_StLB037 : ---LDERVLYAETALRMMGLV---DDFAPIVLLCGHGATVTNNPFASSLQCGACGGHEGEPNARAAAMIFNDPETRRALAAR-GIRIPADTLFLAAQMD :
Micau_3943|Micromonospora_aurantiaca_ATCC_27029 : ---LDEQVYYAEATLRTVGLT---TGFAPLVLLCGHGATSTNNPYAAALDCGACGGNRGGVSARLVAALLNRPEIREALVAR-GIHLPADTHVLAGEHD :
BA_3182|Bacillus_anthracis_str._Ames : GFTKEEKVNYVRQTLKMVGLT---EGFAPLVVMCGHSSQSTNNPYAAALECGACGGAAGGFNARVFATLCNLPEVREALSAE-GIKIPDDTIFAAAEHK :
HD73_2817|Bacillus_thuringiensis_serovar_kurstaki_ : GFTKEEKVNYVRQALKMVGLT---EKFAPLVVMCGHSSQSTNNPYAAALECGACGGAAGGFNARVFATLCNLPEVREALFAE-GINIPKDTIFAAAEHK :
BSU01845|Bacillus_subtilis_subsp._subtilis_str._16 : GFTDEEKVNYARQALKMMGLT---ENIAPLVVICGHGSQSTNNPYSAALDCGACGGAAGGFNARVLAALCNLSEVREALLAE-GIKIPEDTVFAAAEHN :
SA0412|Staphylococcus_aureus_subsp._aureus_N315 : GFTEQEQIDFALQALKLMDLT---EAFAPFVVLAGHASHSHNNPHHASLECGACGGASSGFNAKLLAMICNRPNVRQGLKQS-GVYIPETTVFAAAEHH :
Theco_3474|Thermobacillus_composti_KWC4 : GFSLEEKVNYVGSLLRSIGLT---SAFSPLVVVCGHKSETANNPYASALDCGACGGAAGGLNARVFAELCNREEVRRALAGQ-GIVIPEETVFIAAEHS :
Tmar_0818|Thermaerobacter_marianensis_DSM_12885 : GMRTEEMVQAVKSLFLSIGLV----SFAPLVVVCGHRSLSTNNPYAAALECGACGGAAGGFNARVFAALCNRRDVREGLARE-GLRIPDETVFVAAEHV :
GTCCBUS3UF5_5570|Geobacillus_thermoleovorans_CCB_U : GLTKEEQVQYVKQLLVNIGLT---SSFAPLVVVCGHESETTNNPYASALDCGACGGAAGAFNARVFAALANLPHVRDGLAKE-GIVIPDETVFVAAEHI :
LPE509_03005|Legionella_pneumophila_subsp._pneumop : ----QEQIAYAEMALRLMGLT---DNFAKLVIFCGHGSSTQNNPYASALDCGACGGNQGGKNAQLLASILNKITVRRALAEN-GINIPQDTVFCGAQHD :
Slin_0816|Spirosoma_linguale_DSM_74 : ----ADQCAYAEGALRVMGLT---HHFAPLVVFCGHGSTTQNNAYATALDCGACGGRHGAPNARILAGILNNPEVRTYLVQQ-GIAIPDTTRFIAAEHN :
SNE_A16250|Simkania_negevensis_Z : ----HARTDHAETFLCSIGLS---KHFSKHIFVCGHTSQTENNPYAAALKCGACSGNGGGTNAQTIVAILNDKTVREELKSR-GINIPQDTRFIACEHN :
Belba_2581|Belliella_baltica_DSM_15883 : ----EEKVSIVKSAFDLMGWK---T-FSPIILFVGHGSHTANNAFASSLDCGACAASPGRHNARMLALLANQPDVRLELK-KLNIHIPETSFFMGAEHN :
P700755_003031|Psychroflexus_torquis_ATCC_700755 : ----KEKAAIVKSAFALMGWE---Q-FSPLILFVGHGSHSANNPFGSSLDCGACAASPGRHNARMLAKLANLPEVRTLLKDKEGITIPKDTVFIGAEHN :
Aeqsu_2834|Aequorivita_sublithincola_DSM_14238 : ----DEKVAIVKGAFDLTGWK---T-FAPVVLFVGHGSHTANNPFGSSLDCGACAASPGRHNARMFAKLANLEDVRKTLMEKYAIEIPPDTVFIGAEHN :
DDD_3132|Nonlabens_dokdonensis_DSW-6 : ----ADKVAIVKGAFDLTGWK---K-FAPLVVFAGHGSHTANNPFGSSLDCGACAASPGRHNARMLAKLANLKEVRAELAAQHQIVIPKETVFVGAEHN :
ZPR_2927|Zunongwangia_profunda_SM-A87 : ----EEKVSLVKATFDLTGWK---I-FAPVIIFTGHGSHTSNNPFASSLDCGACAGNPGRHNARTLASIANEKEVRLALKNNFGIDIPENTIFLGAEHN :
SRM_02272|Salinibacter_ruber_M8 : GLADEAKVLYAEAAFRLMGWT---DTFAPVVVFTGHGSQTPNNPYKASLDCGACAGNPGGPNARVLAAICNEDAVQEALRER-GIAIPDDTVFLAGQHN :
Htur_0297|Haloterrigena_turkmenica_DSM_5511 : GMTLEEKVEYAATAFELMGWT---E-FARLVVFAGHASETTNNPFDSSLDCGACAGNPGGPNARVLAEICNDEDVRAELCER-GIDVPEDTVFLAGEHN :
NJ7G_0232|Natrinema_sp._J7-2 : GLSHEAKVEYAQNAFELMGWT---E-FARLVVFAGHASETTNNPFGSSLDCGACAGNPGGPNARVLAAICNDDDVRATLRER-GIDIPEDTVFLAGEHN :
Halxa_1627|Halopiger_xanaduensis_SH-6 : GMSLEQKVGYAENAFALMGWT---E-FARLVVFAGHASETTNNPFDSSLDCGACAGNPGGPNARVLATICNDPDVQAELRER-GFHIPDDTVFLAGEHN :
Natoc_0177|Natronococcus_occultus_SP4 : -MSDDEKVEYAQTAFELMGWT---E-FSRLVVFAGHASHTTNNPFDSSLDCGACAGNPGGPNARILAKICNDESVKAELRQR-DFSIPEDTVFVGAEHN :
NP5058A|Natronomonas_pharaonis_DSM_2160 : -MTHEEKVEYAQTAFELMGWT---E-FARLVVFTGHTSHTTNNPFDSSLQCGACAGNPGGPNARVLAKICNDEAVKDDLRER-GVDIPDDTVFVGAEHN :
Hqrw_2948|Haloquadratum_walsbyi_C23 : GLTHQERVEYAASAFELMGLK---T-FGRVVGFIGHASQTANNPFGSSLDCGACAGNAGGPSARVLAQICNDDAVKSSLRDR-GIDIPVDTVFIAGEHT :
Hmuk_0931|Halomicrobium_mukohataei_DSM_12286 : GLTVEEKVEYAATAFELMGWE---Q-FARLVVFTGHASQTANNPFDASLDCGACAGNPGGPSARVLAAVCNDDAVRERLRDR-GIDVPEDTYFLAGEHN :
HTIA_0481|Halorhabdus_tiamatea_SARL4B : GLTHEEKVEYAETAFELMGIE---A-FGRLVVFVGHASETTNNPFDSSLDCGACAGNPGGPNARVLAAICNDPAVQTALADR-GIDVPGDTVFLAGEHN :
HVO_2410|Haloferax_volcanii_DS2 : GLTDDEKVEYAANAFGLMGWE---E-FGRLVVFTGHASETANNPYDSSLDCGACAGHPGGPNARVLAAICNDETVKAQLRDR-GFGIPEDTVFVAGEHN :
pNG7034|Haloarcula_marismortui_ATCC_43049 : GLTTDEKVEYAATAFDLMGWE---A-FSRLVVFTGHASETTNNPYDSSLDCGACAGNPGGPNARVLATICNDTEVQSALRDR-GFEIPEDTVFMAGEHN :
Hbor_38520|Halogeometricum_borinquense_DSM_11551 : GLTHEEQVEYAATAFDLMGFK---E-FSRLVVFTGHASETANNPYDSSLDCGACAGNPGGPSARVLAKVCNDEAVRAELRDR-GFDIPEDTIFVAGQHN :
Nham_4323|Nitrobacter_hamburgensis_X14 : GYSLDEQVNYVHTALTMIGLT---KTFSRFVLIVGHSGQTENNPYESALDCGACGGASGLVNARVLAQMANKTAVRERLRGM-GIDIPDDTWFLPALHN :
Nham_4329|Nitrobacter_hamburgensis_X14 : GYSLDEQVNYVHTALTMIGLT---KTFSRFVLIVGHSGQTENNPYESALDCGACGGASGLVNARVLAQMANKTAVRERLRGM-GIDIPDDTWFLPALHN :
THI_0132|Thiomonas_arsenitoxydans : GYSLDEQVNYVHTALTMIGLT---QTFSRFVLIVGHQGQTENNPYESALDCGACGGGSGLVNARVLSQMANKTAVRERLATM-GITIPEDTWFLPAVHT :
Tbd_2653|Thiobacillus_denitrificans_ATCC_25259 : GFTLDEQVHFVGQALRSIGLV---SGFSRFVLLTGHGSTSENNPYESALDCGACGGNHGITNARVLAQIANKTAVRARLREQ-GIVIADDTWFVPAFHN :
Hneap_0907|Halothiobacillus_neapolitanus_c2 : GFSLDEQVFYVDKALTSIGLT---ENFSRFVLLAGHGSTSDNNPYESALDCGACGGSHGLVSARVLAHMANKPEVRRRLAKQ-GIQIPEDTWFVSVMHN :
Marky_1969|Marinithermus_hydrothermalis_DSM_14884 : GFSREEQAALVANTLRSIGLT---QGFAPLVLVLGHGSRSENNPYESALDCGACGGAAGTHNARVFCFMANHPKVRELLKQKHGISIPETTHFVPGLHN :
aq_863|Aquifex_aeolicus_VF5 : GFTLDEQASLIGRALKMVGLT----EFAPFVFIIGHGSKSDNNPYESALDCGACGGASGLYNAIVFCRMANNPEVRKRIKEKFGINIPENTYFVPGLHN :
HTH_1102|Hydrogenobacter_thermophilus_TK-6 : GFTKEEQAFLVSTTLKSIGLV---KDFAPIVFVLGHESRSENNPYESALDCGACGGASGIYNARIFCTMANDPAVRQIMKQKHGLNIPEETVFLPGIHN :
Thal_0253|Thermocrinis_albus_DSM_14484 : GFTKEEQAFLISTALKSIGLT---KEFAPIVLVLGHESRSENNPYESALDCGACGGASGIYNARIFCIMANDHVVRQIMAQRYGLEIPPYTVFIPGVHN :
HY04AAS1_0547|Hydrogenobaculum_sp._Y04AAS1 : GYTEEEQIKLVENFLKLIGLT---ENIPKFVLLIAHGSTSDNNPFESALDCGACGGNNGLPNVRILASIANRNQIRKGLEKV-GIKIPEDTIFIPGIHN :
SULAZ_0653|Sulfurihydrogenibium_azorense_Az-Fu1 : GYTLDEQVILAENFLRLIGMV---EDFPEFVLLVGHGSVSDNNPYESALDCGACGGNSGYHNVRAMCMILNKKEVR---EKL-SIRIPDGTIFIPGLHN :
LFML04_2276|Leptospirillum_ferriphilum_ML-04 : GLTPSEQVAFAETALSLAALH---DNFAPLVVFFGHKSTSDNNPYESALDCGACGGQDGSPNARVAASLLNRPSVRAGLEKK-GIRIPDTTWFLAGVHD :
NIDE1770|Candidatus_Nitrospira_defluvii : GFSVAEQAYGVEAALRLMGFT---SGFSRLVVMCSHGSTSDNNPYESALDCGACGGNSGLPNARAFASMANNPAVRKVLESR-GIKIPGDTHFVAALHD :
NIDE4388|Candidatus_Nitrospira_defluvii : GFTLEEQVLTVETALRMMGLV---RNFARLVLFCAHGSTTENNPFESALDCGACGGNEGKPNARVLAAMANRPPVRERLAKR-GIEIPSDTHFLAGQVD :
TPY_2995|Sulfobacillus_acidophilus_TPY : ----EETADLVAAALTGLGIA---RRQGRLVVLLGHRGHSDNNPWASALQCGAAGGHPGGANARALAWLANQTAIRHRLTAR-GIELSPDTWFLAGEHN :
Runsl_0785|Runella_slithyformis_DSM_19594 : GFTIDEMAIRVENLLKSIGLV---DHFAPIVYVIGHGSSSVNNPHYAAYDCGACAGRAGSVNSRVISFMANHPKVRAILSER-GIVIPADTQFVGGLHD :
Slin_1124|Spirosoma_linguale_DSM_74 : GYSVDEMAVRVEGLLKSIGLV---EDFASVVYVVGHGASSVNNPHYAAYDCGACSGRAGSVNARAISYMANHAKVRAILSAK-GLTIPDTTQFIGALHD :
Halhy_2331|Haliscomenobacter_hydrossis_DSM_1100 : GYSIDEMVTRVETVLRSIGLI---KDFAPLVYIVGHGASSVNNPYYAAYDCGACSGRAGSVNARVFSYMANHPEVRSKLAEK-GLLIPAETQFVGALHD :
Emtol_1186|Emticicia_oligotrophica_DSM_17448 : GFTVEEMALRVGNLLNSIGLV---KDFAPIVYVVGHGASSVNNTHYAGYDCGACSGRPGSINARVISFMANHAGVREMLRKQ-GIDIPSTTQFVGALHD :
Avin_17870|Azotobacter_vinelandii_DJ : GFSVAEMAERAENLLGSIGLT---QDFAPIVYVVGHGASNTNNPHYAAYDCGACSGRPGSVNARVICFMLNHPEVRAILAGK-GIEIPAATQFVGALHD :
Nmul_A0708|Nitrosospira_multiformis_ATCC_25196 : GFTIDEMVQRVQGVLTSTGLNGVDKTFAPIVYMIGHGASSVNNPHYTAYDCGACGGRPGSVNARTFCYMANHPKVREALKER-GIIIPPTTQFLPGLHD :
Halhy_3199|Haliscomenobacter_hydrossis_DSM_1100 : GFTVEEMTTRVEGQLRGMGLI---KNFAPIVYIIAHGSSSANNPHHGAHDCGACSGRPGSVNARVFAAMANHPEVRARLHAK-GIDIPAETQFVGGMHD :
P700755_000908|Psychroflexus_torquis_ATCC_700755 : GYTVEEMVEIVYGLLKSTGLT---TNFAPLVYLIGHGGSSTNNPYYAGYNCGACSGRAGSVNSRAVAEMANREDVRKALEAK-HICIPSTTYFLGGLHD :
Metme_3843|Methylomonas_methanica_MC09 : GFSVPEMAEKVGGQLRSIGLI---KNFSQLVVAVAHGSSSVNNPHFAAYDCGACAGKPGAPNARAFAWMANHAGVRAILREQ-GIDIPESTRFAAAMHN :
Turpa_2600|Turneriella_parva_DSM_21527 : GYTVTEMAERVGRVLTQIGLR---QNFAPLVAIVGHGSSSANNPFFAAYDCGACSGRPGLANARVFALMANRTDVRQILAAE-GLAIPEGTHFVGALHD :
STAUR_4541|Stigmatella_aurantiaca_DW4 : GYSLDEQANIVEGALRTIGLT---RAFAPVVAVVAHGSTNTNNPFRQAYGCGACSGNPGEPNARAFALMANRPEVRERLAAR-GLQIPATTLFVPCYHD :
Metme_0526|Methylomonas_methanica_MC09 : GFTDSEQAERIAAFLKNTGLT---YGFAEIVVLMGHGSMSQNNPHLAAYDCGACSGRHGGPNARLFAAMANRAEIRQLLLEH-NIDIPEHTWFIGAEHN :
Nhal_2896|Nitrosococcus_halophilus_Nc_4 : GFTDAEQAAQVAAVLRASGLT---YGFAPLVLLAGHGSISQNNPHMAAYDCGACSGRHGGPNARVFAAMANRPEIRALLAEQ-GIKIPDDTWFIGTEHN :
MCA0512|Methylococcus_capsulatus_str._Bath : GLTDAEQADRVAAFLLNIGLT---QGFAPIVVLMGHGSASRNNPHLAAYDCGACSGRHGGPNARVFAAMANRPQVREQLAQR-GLVVPADTWFVGAEHN :
Nit79A3_1613|Nitrosomonas_sp._Is79A3 : GFTDLEQVERLSVFMRTTGLS---YGFAPIVCLSGHGSTNLNNPHEFGYNCGACSGKRGGPNARLFAAMANRPEIRKLLAER-GINIPDDTWFIGAEHD :
CAP2UW1_3861|Candidatus_Accumulibacter_phosphatis_ : GFSEDEQAARVAGFLRTIGLT---RDLAPLVVIVGHGSDSRNNPHLAAYDCGACSGRHGGPNARVFAAMANRPAVRAGLAVQ-GIVVGDGTHFIGAEHN :
Daro_0565|Dechloromonas_aromatica_RCB : GLTDAEQIERVEAFLRMIGLT---AGFAPLVLMFGHGSGSQNNPHLSAYDCGACSGKHGGPNARVFAAMANRPAVRAGLAAR-GLSIPDSTWFVAAEHN :
SCE1572_25475|Sorangium_cellulosum_So0157-2 : PFSAAEKAARVAATLEIMGLT---RRFAPVVAVLGHGASSVNNPHQSAYDCGACGGRHGGPNARLFAAMANDQEVRALLRGR-GIDIPDGTFFLGGMNN :
SCE1572_28620|Sorangium_cellulosum_So0157-2 : PFTAAEKAARVAVTLENMGLT---RGFAPIVAVLGHGSTSVNNPHQSAYDCGACGGRHGGPNARLFASMANDPEVRALLRGR-GIDIPDGTCFVGGMNN :
Plabr_3227|Planctomyces_brasiliensis_DSM_5305 : GYSLDEMADVVVRLLEDLGLTQT-EQFSRLFIMCGHGSSSLNNPHESAYCCGACAGKRGGPNARAFAMMANDWRVRRLMAER-GIELPEDTFFLGAYHN :
RB10314|Rhodopirellula_baltica_SH_1 : GYSLDEMSQIVVRILQDIGMV---DAFPPIIVFFGHGSGSLNNPHESAYNCGACSGGRGGPNARAFAVMANDPRVRRRVAEQ-GIELPDEVRFVGAYHN :
Sinac_5055|Singulisphaera_acidiphila_DSM_18658 : GYTLEEMTAVGIRQLQDIGLT---SGFARLVVLLGHGSDSLNNPHNSAYNCGACGGAAGGPNARAMAQILNDPRVREGMAKR-GLTLPDETVVVGGYHN :
Psta_3079|Pirellula_staleyi_DSM_6068 : GFSVAEMANMGEKMLRDIGLT---ENFARLVVFMGHGSFCLNNPHKSAYDCGACSGGAGGPNARALATFLNDPRVREIIASR-GLTIPKETWFLGGLHN :
Franean1_5483|Frankia_sp._EAN1pec : GFTVEEMAEIVDTLLTTIGMS---GPLGPVVFVIGHGSSSVNNPHAAAYDCGATGGGQSGPNARAFAAMANHPRVRAALAHR-GRLIGPDTWFVGGHHD :
Hoch_4410|Haliangium_ochraceum_DSM_14365 : GFTVAEMIDIVAGMLRTTGVN---DELAPLFLVIGHGSSSLNNPHEAAYDCGACGGGRGGPNGRAFAMMANDPRVRHGLREQFGLEIPEETWFVGGYHN :
BN6_40270|Saccharothrix_espanaensis_DSM_44229 : -----EMTDIVSTVLRTTGLS---RGLAPVVLIVGHGSSSLNNPHESAHDCGATGGGRGGPNARAFAAMANDPAVRRGLAER-GTVIPEGTWFVGGYHN :
Tcur_2598|Thermomonospora_curvata_DSM_43183 : GYTVPEMTRIVSTVLRTIGLT---GGLAPLVLIVGHGSSSLNNPHEAAHDCGAAGGGRGGPNARAFAAMADHPGVRRALRRE-GLHIPDDTWFVGACHD :

BLOCKS : BBBBBBBBBBBBBBBBBBBBBBBBBBBBBBBBBBBBBBBBBBBBBBBB---------------------------BBBBBBBBBBBBBBBBBBBBBBBB :
SITE : X----------------------------------------------------------------------------------XXX------------- :
LFML04_1811|Leptospirillum_ferriphilum_ML-04 : TTTDMVALYDKDT-LPPSH---DGDIRHLEQWLEQAGRLTRMERSVFLGT-----------GDLSSEDVFADVRRRTRDWSEVRPEWALAGNAAFIAAP :
LFML04_1818|Leptospirillum_ferriphilum_ML-04 : TTTDDVELKDTDT-LPPSH---REDLRRLEKWLEEAGELARMERSVLLGL-----------REKDTRTVETDIRRRTRDWSEVRPEWGLAGNAAFIAAP :
AFE_1661|Acidithiobacillus_ferrooxidans_ATCC_23270 : TTTDEVTIFDTED-LPTTH---AKDLAQLRQWLADAGELTRLERATLLGT-----------ASQAPEVVTRDMRRRTRDWAEVRPEWALAGNAAFIAAP :
Afer_0133|Acidimicrobium_ferrooxidans_DSM_10331 : TLTEQVRLFDVDA-LDPAH---RTLARDLETDLASATRLAATWHATSLGV-----------T--ADQDLAHDLARRGRDWAEVRPEWGLVANAAFIAAP :
Thimo_2946|Thioflavicoccus_mobilis_8321 : TTTDTLRLFDTDE-VPAAL---AQDLAQLRQWLEQAGGLTRMERATLLGI-----------AGLPDRAVEADVHQRSRDWSQVRPEWALANNAAFIAAP :
THI_0793|Thiomonas_arsenitoxydans : TTTDIVQVFDAHA-VPHDL---APDLANLERALEQAGDLTRMQRASSMGI-----------DAMTDRDVAQHVQARSRDWSQVRPEWALANNAAFIAAP :
Hneap_0211|Halothiobacillus_neapolitanus_c2 : TTTDHIELLDLDQ-SGIE----SDQLSSLTQALKQAGELTRLERLVTLEA-----------QV-DTVDAEKQATFRGRDWSQVRPEWGLAGNAAFIAAP :
Ftrac_3394|Marivirga_tractuosa_DSM_4126 : TSTDEISLFNANL-VPSSH---QADLENIRKRIAQAGSATRSERVLRMNI-----------E--DGKDVDSFMKIRAKDWSQVRPEWGLAGCSSFVVAP :
S58_63940|Bradyrhizobium_oligotrophicum_S58 : TTTDAVTLFDEDD-VPAGL---APDLAQLKARLADAGRLARRERSALLGI-----------R--DAVDVDGALIARSRDWSQVRPEWGLAGNAVFIAAP :
Cagg_2891|Chloroflexus_aggregans_DSM_9485 : TTTDDVTIFDKGD-IPASH---ADDLQRLERDLVAAGRLARAERAALLNV-----------D--RNTDIDRAVRRRSTDWSQVRPEWGLAGCAAFIAAP :
RoseRS_3853|Roseiflexus_sp._RS-1 : TTTDDVTIFDKAH-IPASH---TADLKRLEADLAAAGRLARAERSMLLKI-----------G--TKTDIDSAVRRRSKDWSQVRPEWGLAGCAAFIVAP :
SPICUR_00740|Spiribacter_sp._UAH-SP71 : TTRDRVQLYDQDL-ETAVD---RDLLIRVQTWMADAGELARTERGRSL----------------PHAESTRAIERRARDWSEVRPEWGLAGCQMLIAAP :
Dshi_0997|Dinoroseobacter_shibae_DFL_12__DSM_16493 : TAQDAITLYADDL-SEAHRAAATASLAQARQWCAEAGRLARSERQPSL----------------PGATERDGIAARAQSWAETRPEWGLAGCKAFVVAP :
Gal_04259|Phaeobacter_gallaeciensis_DSM_26640 : TTTDQVTLYEQDL-PTGCETRLARDLARLRRWVKAAGALARSERAARL----------------PRAKAGGDILRRSSDWAELRPEWGLAGCRAFVAAP :
Jann_1258|Jannaschia_sp._CCS1 : TTTDAITLFAKDH-PSAAH---DAGIAQAETWFAQAGTVTRAERALRL----------------PRADGDADVDLRSRDWAETRPEWALAGCKAFIAAP :
RLO149_c003320|Roseobacter_litoralis_Och_149 : TTTDAVTLYEADH-PSKAH---AADLKQAKAWFLSAGSVTRAERALRL----------------PRADGTDDIALRSRDWAETRPEWALAGCKAFVAAP :
ACMV_25640|Acidiphilium_multivorum_AIU301 : TTTDDVTLYLDDH-PSAAH---RGDLDQARLWLTSAGKIARTERALRL----------------PRAASDASLPKRGRDWSETRPEWALAGCQAFIAAP :
RPA2996|Rhodopseudomonas_palustris_CGA009 : TTTDAVTLYDADH-PSPAH---ASALAQTRDWLATAGALTRSERALRL----------------PRAATGGAIARRARDWAEVRPEWALAGCRAFIAAP :
PXO_00907|Xanthomonas_oryzae_pv._oryzae_PXO99A : TTTDAVTLFADDH-PSPTH---AQDLAQVTQWLAAAGALARGERALRL----------------PRANRSQDIAHRARDWAEIRPEWALAGCQAFIAAP :
RC1_1188|Rhodospirillum_centenum_SW : TTTDEVTVYAADH-PSVAH---AGDLERARRWLTSAGVLARGERALRL----------------PRAARSQDIPHRARDWAELRPEWALAGCQAFVAAP :
AZC_1310|Azorhizobium_caulinodans_ORS_571 : TTTDDVNIYAADH-ASAAH---AEDVDQAERWLKSAGILARGERALRL----------------PRAKQGQDIPHRARDWAELRPEWALAGCQAFIAAP :
HP15_1623|Marinobacter_adhaerens_HP15 : TATDKVTIADRHL-IPDSH---MQHLADLEAGFETAGIRARKERATPLKL-----------NGLDDDNLKQAMETRTRDWSEVRPEWGLANNAAIIFAK :
CV_3220|Chromobacterium_violaceum_ATCC_12472 : THSDEILLLDADT-LSADA---RARLPGLQAQLRAASALARRRRAPLVGL----------APEADDAILLNKMRQKGDDWAETRPEWGLAGNALFIAAP :
NH8B_2409|Pseudogulbenkiania_sp._NH8B : THSDELHLLPASS-PDPAL---RQHAEALRPVLDQVERRARARRAGIDGV----------PDHLDDAELLALLRQKGHHWAETRPEWGLTNNAFFIAAP :
IL0525|Idiomarina_loihiensis_L2TR : TVTDKVIWLSNT--VPDSV---KQVFRAATEKLTQACGESEKDRKTR-----------------------------SGHWAELRPEWGLADNNVLFFGQ :
Mmwyl1_3800|Marinomonas_sp._MWYL1 : TTTDQLTCFDQTK-DAKPI---DRKIKDW---FEKAQFLAQQERAAKLDTALL-------D--ASDKQRSKAFSKRANDWSQVNPEWGLANNHSFIIAP :
Tcr_0854|Thiomicrospira_crunogena_XCL-2 : TTTDEFTCFGLNH-----V---DETIQKW---LARATEFARQERSTRLGLNHL-------E----GQNLHQSIQRRAKDWSQVRPEWGLSNNAAFIVAP :
HP15_4110|Marinobacter_adhaerens_HP15 : TTTDEVECSG--D-----V---PDEIRGF---LANAGAQARRERALRLGIANE-------S------DVDSAIKKRSQDWSEVRPEWGLAGNASFIVAP :
TOL_3715|Thalassolituus_oleivorans_MIL-1 : TTTDHIQCFG--Q-----V---SELGTQW---LKDATVLAQRERAVGLGLADE-------AFFNQPEKLDAAIQRRALDWSEVRPEWGLAGNSAFIVAP :
VC1582|Vibrio_cholerae_O1_biovar_El_Tor_str._N1696 : TTTEAMRLFDRHE-IPTSH---REALEQLDQQLTAASHGARQERAPSLELNHNHQAPPSKDNALSAQQLEQAFLRRAHDWAQTRPEWGLTNNAAFIIAP :
RGE_20630|Rubrivivax_gelatinosus_IL144 : TTTDEVTAFDLDL-HGAPV---HERWARLQDAFDLAADRVRRERAPALGL----------DPRAAQAWLLEDLRRRANDGAQTRPEWGLAGNAAFVVAP :
Hsero_2320|Herbaspirillum_seropedicae_SmR1 : TTTDEVEAFDLDL-LPAPA---QQRWAAMREVFARAGEQVRRERAARLGL----------DGEQPAARLLQALRRRANDGAQTRPEWGLTGNAAFLIAP :
Acav_3940|Acidovorax_avenae_subsp._avenae_ATCC_198 : TTTDEIEGFDLDL-LPPEA---RARWERWQPVFATAGDRVRRERAPSLAM----------DPHAAAGELLSALRRRANDGAQTRPEWGLAGNAAFVIAP :
Msip34_2093|Methylovorus_glucosetrophus_SIP3-4 : TTTDEIEGFDLDL-MPEDA---RARWQTLQQVFAQAGNQVRRERSPSLQL----------DAQASDDALLEQLRRRANDGAQTRPEWGLAGNAAFIIAP :
Alide2_2978|Alicycliphilus_denitrificans_K601 : TTTDEIEGFDLDL-LPTPA---RRRWECLQDVLAHAGDQVRRERAPALQL----------DPRAPHGALLQQLRRRANDGAQTRPEWGLAGNASFVIAP :
U875_08155|Pandoraea_pnomenusa_3kgm : TTTDELEGFDLDL-LPQAA---RDRWARLQPIFAQACDQVRRERAASLTL----------DPRAPHHALLARLQRRANDGAQTRPEWGLARNAAFLIAP :
Isop_0797|Isosphaera_pallida_ATCC_43644 : TTTDEVRLLDLER-LPQSH---RDDLERLAADLKEAGRRVRQERAADLGL-----------ANRPLSLLDRRFKRRAADWSEVRPEWGLARNCAFIAAR :
PB2503_13514|Parvularcula_bermudensis_HTCC2503 : TTTDDITFLPEPL-WPESH---VTDLDKAKAVFSAAGAATRRARAHGLGL--------------TGKTVEPQIRRRSRDWSEIRPEWGLAGCNAFIAAP :
RB1129|Rhodopirellula_baltica_SH_1 : TTTDQIEWLDLDA-VPASH---QSRIVELQNVADAASHLTREERLPLL-----------------NESCTDSLISRASDWSQTRPEWGLAGNASMLIGP :
Plabr_4776|Planctomyces_brasiliensis_DSM_5305 : TTTDDLQLIKDDN-LPEHH---GQDLAELQLLALQAGQQTRLERAGRL-----------------GAISESDVRNRARDWSEIRPEWGLAGNAAFVVAP :
TMO_0417|Tistrella_mobilis_KA081020-065 : TTTDRVAL--DPD-PVAAS---RQSFRRLQADLDRARRAAAAERVSHLTG---------AD----AVDPVRAVEKRAADWSEVRPEWALAGNAAFIVGD :
MTES_1382|Microbacterium_testaceum_StLB037 : TVTDEVTLLEPWA-VPATH---ETAVLELGQYLEAARAADAADRSASLPG---------GSD---AAGAVRDTERRAADWAESYAEWGLAGNAAFIVGP :
Micau_3943|Micromonospora_aurantiaca_ATCC_27029 : TVTDEVRLFDVDT-VPVRL---RPHVDDLTRRLAEAGAGLRAERATRLPG---------RPG---S----RHLPGRASDWAQVRPEWALAGNAAFIAAP :
BA_3182|Bacillus_anthracis_str._Ames : TTVDELEWIYVPE-LSETA---QEAFDCIEAIMPNVSQHANRERLMQLPH---------FKTK--IKNPSKEAHRFAEDWSEIRPEWGLARNASFIIGQ :
HD73_2817|Bacillus_thuringiensis_serovar_kurstaki_ : TTVDELEWIYVPE-LSKTA---QEAFDCIETIMPNVSQHANRERLTQLPN---------FKTK--IKNPSKEAHRFAEDWSEIRPEWGLARNASFIIGQ :
BSU01845|Bacillus_subtilis_subsp._subtilis_str._16 : TTVDELHWLYVPE-LSEAA---QEAFEQIEAVMPKVRHHVNAERLAQLPN---------FQSK--LKNPKAEANRFAEDWSEIRPEWGLARNAAFIIGK :
SA0412|Staphylococcus_aureus_subsp._aureus_N315 : TSTDTLAWVYVPDTLSALA---LDAYESLNDAMPMISEQANRERLDKLPT---------IGRV---NHPVEEAQRFASDWSEVRPEWGLAKNASFIIGR :
Theco_3474|Thermobacillus_composti_KWC4 : TTVHELRWLHVPE-LSPAA---RDAFALLQDRLRAVTRKVNLEQLAKLPG---------AGAA--GRDPVAEAHRRAADWSEVRPEWGLAGNYAFVIGR :
Tmar_0818|Thermaerobacter_marianensis_DSM_12885 : TTLDVLQWVDVPP-LTPAA---QEAFARLLPVLDQVSRRTRAERVVKLPH---------VGAV---RDPHAEACRRATDWSEVRPEWGLAGNAAFVVGR :
GTCCBUS3UF5_5570|Geobacillus_thermoleovorans_CCB_U : TTVDELRWVEVPP-LSEAA---EAAFRQLKQALAGVSRQANAERMAKLPH---------VGAM--PRDPVAEARRRAVDWSEIRPEWGLAGNAAFLIGR :
LPE509_03005|Legionella_pneumophila_subsp._pneumop : TTTDEVEIYHSNV-SQFID---QDILDQLRADLNMAKHNNNLERINYLN------------S---IDCAEKDIVRRSADWSETRPEWGLARNAAFIVAP :
Slin_0816|Spirosoma_linguale_DSM_74 : TTTDEVTLYGDDA-S--------EACKKLTRDLAKAQQANSLERLRQMQK---------NAD---HSGGAQQTWLRSQDWAQVRPEWGLARNAAFIVGP :
SNE_A16250|Simkania_negevensis_Z : TTTDQFTYFLEQD-EKTL------ELQTIIEHLEQACSENRIKRLKQLGV---------KTT---AKTSMRKASLRGQKWSETRPEWGLAKNGSFIIGP :
Belba_2581|Belliella_baltica_DSM_15883 : TTTDEIVIF-DSE-LPDSH---KEQFLQLKENLFKAQVSATAERLNT------------------SKNSIQAAEKKSNNWSETRPEWGLAKNAGFIVGP :
P700755_003031|Psychroflexus_torquis_ATCC_700755 : TTTDEIVMF-DSE-VPNSH---KEKLDRLKIDLHFAQQTATQERLGS------------------GMKSIKAAHKKTNDWSETRPEWGLAKNAGFIVGP :
Aeqsu_2834|Aequorivita_sublithincola_DSM_14238 : TTTDEIVIF-DAD-IPNIQ---NKALQKIKANLAKAQENATAERLNN------------------TKKSISQAQRNANNWSETRPEWGLAKNSSFLIAP :
DDD_3132|Nonlabens_dokdonensis_DSW-6 : TTTDEIELF-DIE-VPASH---KAPLADLKESLKKAQQTAAKNRLGV-------------------KDSVGLAEKKANNWSETRPEWGLAKNAGFIVAP :
ZPR_2927|Zunongwangia_profunda_SM-A87 : TVTDEIEIF-DTE-FAPNH---KEALLKITENLKQAQLEASRERLGS------------------EKESLKLAHKKSHNWSETRPEWGLAKNASFIIGP :
SRM_02272|Salinibacter_ruber_M8 : TTTDEIALFVDED-DPPVA---PDALDRLRRDLHAAQADAATERVRTLNT---------SVDEGRPAAAVRETERRAADWAETRPEWGLAGNAAFIVGP :
Htur_0297|Haloterrigena_turkmenica_DSM_5511 : TTTDEIELF-DNA-VPESH---REDLASLRADLEAARADSAAERTASAGD---------D--------AVGEVERKAADWAEARPEWGLAGNASFVIGP :
NJ7G_0232|Natrinema_sp._J7-2 : TTTDEITLF-DGS-VPESH---REDLASLRGDLERARAGAAAERTASADD---------GA-------AVDEVERKAADWAETRPEWGLAGNASFVIGP :
Halxa_1627|Halopiger_xanaduensis_SH-6 : TTTDEITLF-DGA-VPESH---REDLEQLRADLERAQAGAAAERLESMTD---------ETDVD-PEEAVAEVERKAADWAETRPEWGLAGNASFVIGP :
Natoc_0177|Natronococcus_occultus_SP4 : TTTDEITLF-DGD-VPESH---HEDIEELRDDLEQARRAAAAERSESLTN---------A---D-PSDPIRETARRAVDWGETRPEWGLAGNASFVIGP :
NP5058A|Natronomonas_pharaonis_DSM_2160 : TTTDEITLF-DGE-VPKSH---HADVAALRDSLEQARAGAAAERSEALNG---------A---D-PDKGVSETASRAADWAQTRPEWGLAGNASFVIGP :
Hqrw_2948|Haloquadratum_walsbyi_C23 : TTTDKITLY-TEA-IPDSH---QDDIRSLQADLSIAQEDAAAERLESLSG---------DT----TVDAIQDIERRAADWAETRPEWGLAGNAGFVIGP :
Hmuk_0931|Halomicrobium_mukohataei_DSM_12286 : TTTDEIELY-ADA-VPETH---ADDLDALRADLETARAGAAAERADDMGA---------D-----GDAG-RDTHRRAADWAETRPEWGLAGNAGFVVGP :
HTIA_0481|Halorhabdus_tiamatea_SARL4B : TTTDEVELF-DDE-VPESH---AEDLEKLRADLSVARENAAGERAAAMKT---------D-----DSAGVREIERRAGDWAETRPEWGLAGNAGFVIGP :
HVO_2410|Haloferax_volcanii_DS2 : TTTDEIELY-DGD-VPESH---AEDLDQLRADLAVAREHAAAERAETMGA---------G-----GSAAVSETERRAADWAETRPEWGLAGNAGFVIGP :
pNG7034|Haloarcula_marismortui_ATCC_43049 : TTTDEVELY-DSE-VPESH---ADDLKQLRANLATARENAAAERAESMGS---------D-----ASSGVSETQRRAADWAETRPEWGLAGNAGFVIGP :
Hbor_38520|Halogeometricum_borinquense_DSM_11551 : TTTDEVELF-VDD-VPESH---SDDLDQLRTDLTTARENATTERAEAMGA---------D-----GSTGVTETERRAADWAETRPEWGLAGNAGFVIGP :
Nham_4323|Nitrobacter_hamburgensis_X14 : TTTDAIELSDLVL-LPPRH---LVYLDRLRNGLRAASRLAAAERMPKLLP-----QA----RAIEPAQAWRLAHRLAVDWAQVRPEWGLSKNVYGIIGR :
Nham_4329|Nitrobacter_hamburgensis_X14 : TTTDAIELSDLVL-LPPRH---LVYLDRLRNGLRAASRLAAAERMPKLLP-----QA----RAIEPAQAWRLAHRLAVDWAQVRPEWGLSKNVYGIIGR :
THI_0132|Thiomonas_arsenitoxydans : TTTDSIELLDLDL-LPPRL---LVYLERLRNGLRAASRLAAAERMPKLMS-----SP----RELDPAHAYRLAHRLAVDWSQTRPEWGLSKNVYGIIGR :
Tbd_2653|Thiobacillus_denitrificans_ATCC_25259 : TTTDELRLYDLDL-LPPSH---LVYTERLINGLQAASHLCAAERMRTLQD-----TPGDADENGDSAGAYRLARRNALDWSQVRPEWGLARNAAFVIGR :
Hneap_0907|Halothiobacillus_neapolitanus_c2 : TTTDQLSLQDLDL-LPNSH---LVYLERLRNGLRAATRLSAAERLPALLD-----HPS---PNIDTLSAQKQIERNASDWTQVRPEWGLARNASVVAGG :
Marky_1969|Marinithermus_hydrothermalis_DSM_14884 : TTTDAVQLYDLEY-LPSKL---LPVLEGVRADLEAATLHTAQERALELGV-----KP-----------EYAEVLEHAYDWSQVRPEWGLSGNYAFVIGR :
aq_863|Aquifex_aeolicus_VF5 : TTTDEVHFYDLEQ-FPQEV---KEKLKEIKEDFDKASMLTASERYKELFD-----E-----EAEDELRKIYKVVENAYDWSQVRPEWGLSGNYAFVIGR :
HTH_1102|Hydrogenobacter_thermophilus_TK-6 : TTTDQIVLYDLEY-LPARY---VPMLERIRRDFEEARRLTAQERAIALDA-----G------------SPEQVYRKAYDWSEVRPEWGLSGNYAFVIGR :
Thal_0253|Thermocrinis_albus_DSM_14484 : TTTDEVFLYDLEF-LPAEY---IPLIDKIIQDLQVAKDLTLQERAKTLDT-----K------------NTQDVYKKAYDWSEVRPEWGLSGNYAFIIGR :
HY04AAS1_0547|Hydrogenobaculum_sp._Y04AAS1 : TTTDEITFYDTEV-MPQKD---RALFDKIVKDFKIASQKTREERAKTLPY----------------AGSGDRIPVRAIDWSETRPEWGLSKNMGVYVGK :
SULAZ_0653|Sulfurihydrogenibium_azorense_Az-Fu1 : TTTDEIEFYDEDL-VPENS---LDKWEEIKKDFKKAGEKTRIERLSSLPY----------------ADNPEDVIVRSIDWSEMRPEWGLSKNLGVFVGK :
LFML04_2276|Leptospirillum_ferriphilum_ML-04 : TTTDTFTLYDGED-WPVTH---EKAIRELVRDLHQAGLSLAGERLRTFPG----------SYQKDTDDISARLSARSHDWAEVRPEWGLSGNAAFLVGP :
NIDE1770|Candidatus_Nitrospira_defluvii : TTRNDVRIVDLED-VPPTH---RKDLVRLLEDLEEAGTQAALERGLALER---------PSAKPTRRDPQQRASRRSVDWAQVRPEWGLSRNNLLIIGR :
NIDE4388|Candidatus_Nitrospira_defluvii : TTTDEVHLFDLED-APPTH---RKDVARLYDDLREAAQLTSQERCSRFPD---------VGTVLPLNQASAHVAGRSADWSQVRPEWGLSGNTTFIIGR :
TPY_2995|Sulfobacillus_acidophilus_TPY : TTTDEVTLFDRHR-VPASL---RSEVEQLQRDLAEAGRLNAVERSLDLPG---------APAKPSIEAAIRHAYQRSVDWAETRPEWGLASHFAIVIGR :
Runsl_0785|Runella_slithyformis_DSM_19594 : TTRDEIVYFDENS-LSPKN---LTSHNKNEIIFEKALDYNAKERSRRFES---------IDTTLSPQKIHEKIRLRSVSLFEPRPELNHATNALCIIGR :
Slin_1124|Spirosoma_linguale_DSM_74 : TTRDEIAFFDEAS-LTPEN---FEKHKQNKRTFETALDYNAKERSRRFES---------IDSRASLKRIHEKIRERSVSLFEPRPELNHATNALTIVGR :
Halhy_2331|Haliscomenobacter_hydrossis_DSM_1100 : TTRDEIVFYDEFS-LPPAH---FELHTKNEVVFEKALDYNAKERSRRFYT---------VDTRKAPIKVHEKVLRRSVSLFEPRPELNHATNALCIIGG :
Emtol_1186|Emticicia_oligotrophica_DSM_17448 : TTRDEIEFYDTDI-LIEEN---QPKHKNNHTLFVKALDFNAKERSRRFES---------IDTKLSPEEVHERIRRRAVSLFEPRPELNHATNALCIVGR :
Avin_17870|Azotobacter_vinelandii_DJ : TTRDEIAFYDEDS-LSPDS---RARHRANAAVFDKALALNAKERSRRFEL---------TDSQQSPERVHEAVKARAVSLFEPRPELNHATNALCIVGR :
Nmul_A0708|Nitrosospira_multiformis_ATCC_25196 : TTRDEVAFFDEEI-LSEEN---AGKHRRNTLVINEALDLHAKERSRRLVS---------IDTKMSPKEIHEEIRRRSVSLFEPRPELNHATNAVSIIGR :
Halhy_3199|Haliscomenobacter_hydrossis_DSM_1100 : TAADEMGYFDVRL-LTAEN---FGLHRKNAAAFEKALDLNAKERSRRFAS---------INSKMRIDKIRKAIKDRSVSLFEPRPELGHGTNTLCIVGR :
P700755_000908|Psychroflexus_torquis_ATCC_700755 : TTRDEVTFYDEVT-LSRKL---TEAHDTHKAQFDKALSHNAKERSRQFLL---------TNSKRDAIKVHNEVKNRSTALFEPRPELNHSNNTLCIVGR :
Metme_3843|Methylomonas_methanica_MC09 : TSRDEMVYFDQQS-LDKQT---HNILHNFQHAAHKALQRNARERCRWFEL---------GPQTPDNDAAHRHVLERAASIFEPRPEYNHSNNLYCIVGR :
Turpa_2600|Turneriella_parva_DSM_21527 : TTRDEILFLDENQ-IPATH---TAALSDLKKNFATALKNTALERSRRFED---------VPFQKSAESAQKECIARSEMLFEPRPEYTHATNSVGIVAP :
STAUR_4541|Stigmatella_aurantiaca_DW4 : TSLDAVEVLDRDR-LPAER---LADVAELEARMGRACRMNAVERCQRFGQ---------GP-RAGAEAAAQHVLDRGHDLSQPRPEYGHNRVAACIVGR :
Metme_0526|Methylomonas_methanica_MC09 : TCDEDITWYDSAD-IPAAR---LPAFQRFVAEMEHAQRMSAHERCRRLAS---------APRKPTPAQALRHFLNRAADFSQARPELGHATNAAALVGR :
Nhal_2896|Nitrosococcus_halophilus_Nc_4 : TCSEEFFWYDLGL-LPADF---KPAYEKLKAEIDHALLFSAHERCRRLAS---------APRRPSLQKAMKHIVERSTDFSQVRPELGHATVCWAHIGR :
MCA0512|Methylococcus_capsulatus_str._Bath : TCSEEITWFDVQD-IPPLS---RIGWHAFAAQLDEACRLSAQERCRRFAS---------APRRPSPARALRHVSTRSVDFSQARPELGHATNAAALIGR :
Nit79A3_1613|Nitrosomonas_sp._Is79A3 : TCSDEFFWFDLED-IPQSS---LPAFETFRNDLVKASKISAQERCRRFVS---------ANNPQTPEEGKEHVNLRANDFSQAFPEFNHATIAAAIVGR :
CAP2UW1_3861|Candidatus_Accumulibacter_phosphatis_ : TCDETFVWYDVDR-IPGSH---REAFAALRRDCEQAARLHAVERCRRFAS---------APREPSPQRAQRHLADRRQDIGQARPELGHATVAAAFVGR :
Daro_0565|Dechloromonas_aromatica_RCB : TCDDGIEWYDLDS-TPERF---QAAVDRLLGQMAEACRAHAAERCRRLAS---------APFKPSPWKARQHMIGRANDISQARPELGHATNAAAFIGR :
SCE1572_25475|Sorangium_cellulosum_So0157-2 : TTTEEIVFYDEHL-APASH---RGEIEALEQALGEARKRHAHERCRRFAS---------APERLSPEQALAHVEARAVDLSEARPELGHATNAVCVIGR :
SCE1572_28620|Sorangium_cellulosum_So0157-2 : TTTDEIVLYDRHL-APASH---RAELAALSGALDAARALHAHERCRRFAS---------APAEGGPARALAHVESRAADLSEARPELGHVTNAVCVVGR :
Plabr_3227|Planctomyces_brasiliensis_DSM_5305 : TCDDSVVFYDLDR-LPASH---HADFDAARNDIETARQRNAHERCRRFES---------VPTTVTPLDALRHVEARAQDIAQARPEYNHATNALCVVGR :
RB10314|Rhodopirellula_baltica_SH_1 : TCNDDVDYYDLDL-LPRSL---RELFRRIESDVIETRARNAHERARRFES---------APLDLTPQEALEHVEERAEDLSQARPEYNHATNALVTVGR :
Sinac_5055|Singulisphaera_acidiphila_DSM_18658 : TCNESVIFFDLDC-LSDSH---KAEFATVRKLIEEVCNRNAHERCRRFMS---------APLSISFEAAREHVEERSEDLAQTRPELGHATNAITIVGR :
Psta_3079|Pirellula_staleyi_DSM_6068 : TALDSVTFSNLDL-LPASH---RRDLESMTKTLEEACGRNAHERCRRFYS---------APLDMTPSQARTHAEERSEDLAQTRPEFGNASNAMCFVGR :
Franean1_5483|Frankia_sp._EAN1pec : TCDSSLAYYDTDL-VPAHL---RPALTAATDALLTAVQLDAHERCRRFES---------VGPDVAAGTAHAHVRGRSEDIGQSRPEYGHSTNATCVIGR :
Hoch_4410|Haliangium_ochraceum_DSM_14365 : TCDDSIVYYDVEL-VPERL---RGELAAIQKTLDEVRRLDAHERCRRFED---------APLKMSAERGLANAEAHAVDLGQARPEYCHATNAICFVGR :
BN6_40270|Saccharothrix_espanaensis_DSM_44229 : TCDDTMTYYDEDL-VPAGH---AEPLRAAKVALAEACALAAHERCRRFES---------APPDLAPEDALAHAETHAVDLGQPRPEYGHATNAVCVVGR :
Tcur_2598|Thermomonospora_curvata_DSM_43183 : TCTDTITYYDEDL-VPERC---RGALRAAKRAMARACTLTAHERCRRFES---------APADLPIGRAPAHVAGRAVDLGQPRPEYGHATNAVCIVGR :

BLOCKS : BBBBBBBBBBBBBBBBBBBBBBBBBBBBBBBBBBBBBBBBBBBBBBBBBBBBBBBBBBBBBBBBBBBBBBBBBBBBBBBBBBBBBBBBBBBBBBBBBBB :
SITE : ----------------------X----------------------------------------X--------------------------X-------- :
LFML04_1811|Leptospirillum_ferriphilum_ML-04 : RSRTASLNLGGRAFLHDYDWQRDKD--FATLQLIMTAPMVVGNWINMQYYGSMVDNLHFGSGNKVLHNVVGGSIGVLEGNGGDLRTGLAIQSLHDGH-R :
LFML04_1818|Leptospirillum_ferriphilum_ML-04 : RSRTASLNLGGRAFLHDYDWQRDKD--FATLQLIMTAPMVVGNWINMQYYGSMVDNLHFGSGNKVLHNVVGGSIGVLEGNGGDLRTGLAIQSLHDGH-R :
AFE_1661|Acidithiobacillus_ferrooxidans_ATCC_23270 : RQRTRGVDLEGRAFLHDYDWHKDAG--FSTLELIMTAPMVVANWINMQYYGSMVDNLRFGSGNKVLHNVVGGSIGVLEGNGGDLRVGFALQSLHDGK-R :
Afer_0133|Acidimicrobium_ferrooxidans_DSM_10331 : RARTAHVSLEGRAFLHDYDWRHDTD--FATLTLIMTAPMVVANWINLQYYASMVDNRRFGSGTKVLHNVVGGSIGVLEGNNGDLRVGLALQSLHDGT-R :
Thimo_2946|Thioflavicoccus_mobilis_8321 : RARTTGMDLGGRAFLHEYVWQKDED--FRILELIMTAPMVVANWINMQYYGSVVDPRRFGSGNKVLHNLVGGAIGVLEGNSGDLRVGLPLQSLHDGR-R :
THI_0793|Thiomonas_arsenitoxydans : RFRTRGADLGGRAFLHDYVWKNDAD--FKVLELIMTAPMVVANWINMQYYGSVVDNSRFGSGNKVLHNVVGGAIGVLEGNGGDLRVGLPLQSLHDGS-R :
Hneap_0211|Halothiobacillus_neapolitanus_c2 : RWRTRGLDLGGRAFLHDYDWRHDKE--FGVLNVIMTAPLIVANWINLQYYGSTVDNLHQGAGNKVLHNVVGGTVGVIEGNGGDLRVGLAMQSLHDGE-Q :
Ftrac_3394|Marivirga_tractuosa_DSM_4126 : RTITRGVDLKGRSFLHSYSWREDRG--FKVLETIMTAPMVVTSWINLQYYGSTVDNKHFGSGNKTLHNVTSG-IGVLEGFAGDLRSGLPMQSIHDGV-N :
S58_63940|Bradyrhizobium_oligotrophicum_S58 : RSFTRGLDLGGRAFLHSYEAGRDDG--HRTLELIMTAPMVVASWINLQYYGSTVNNAAFGSGNKVLHNIV-GQLGVLEGNAGDLRSGLPWQSVHDGT-R :
Cagg_2891|Chloroflexus_aggregans_DSM_9485 : RERTAGISLDGRAFLHNYNWRQDGD--FSVLELIMTAPMIVASWINLQYFGSTVDNRVFGSGNKTLHNVV-GTLGVLEGNGGDLRVGLPWQSVHDGK-R :
RoseRS_3853|Roseiflexus_sp._RS-1 : RDRNAGVVMNGRSFLHSYEWRQDEG--FGVLELIMTAPMIVASWINLQYYGSTVDNRVFGSGNKTLHNVV-GTLGVLEGNAGDLRVGLPWQSVHDGE-K :
SPICUR_00740|Spiribacter_sp._UAH-SP71 : RTVTRGIDLGGQAFLHDYDWRADAGQDYPVLELILTAPVIVASWISLQYYGSAVAPALFGAGNKLIHNIVGG-IGVLEGNGGMLRTGLPWQSVHDGE-G :
Dshi_0997|Dinoroseobacter_shibae_DFL_12__DSM_16493 : RTQTAPAQLDGRVFLHSYDWAQDEG--FGVLELILTAPVVVASWISLQYYGSVVAPEVFGGGSKQVHNVTGG-MGVLDGGTGALRIGLPIQSVHDGG-S :
Gal_04259|Phaeobacter_gallaeciensis_DSM_26640 : RSRTEGTALDGQSFLHSYDWRADEG--FGVLELIMTAPVVVASWISLQYYGSSVAPALFGGGNKLLHNVAGG-IGVLEGNGGALKPGLPWQSVHDGE-A :
Jann_1258|Jannaschia_sp._CCS1 : RHRTAGKSLAGRAFLHDYDWKKDSD--FSVLELIMTAPVVVASWISLQYYGSTVAPDVFGSGNKLLHNVTGG-IGVVEGNGGTLRAGLPWQSVHEGE-G :
RLO149_c003320|Roseobacter_litoralis_Och_149 : RQRTAGRSLEGRAFLHDYDWQQDKG--FGVLELIMTAPVVVASWISLQYYGSTVSPDVFGSGNKLLHNVTGG-IGVVEGNGGTLRTGLPWQSVHEGE-D :
ACMV_25640|Acidiphilium_multivorum_AIU301 : RRRTAGKSLGGRAFLHDYDWKQDKS--FSVLELILTAPVVVASWISLQYYGSTVAPDVFGGGNKLLHNVTGG-IGVVEGNGGLLRVGLPWQSVHDGE-R :
RPA2996|Rhodopseudomonas_palustris_CGA009 : RPHTSGRDLQGQAFLHDYDWRKDTD--FSVLELILTAPVVVASWISLQYYGSTVAPETFGAGNKLLHNVTGG-IGVVEGNGGLLRAGLPWQSVHDGE-R :
PXO_00907|Xanthomonas_oryzae_pv._oryzae_PXO99A : RSRSAGRDLAGRAFLHDYDWRCDHG--FGVLELILTAPVVVASWISLQYYGSTVAPERFGAGNKLLHNVTGG-IGVVEGNGGILRTGLPWQSVHDGE-R :
RC1_1188|Rhodospirillum_centenum_SW : RERTAGRDLEGRAFLHDYDWRRDKG--FGVLELILTAPVVVASWISLQYYGSVVAPDVFGAGNKLLHNVTGG-IGVVEGNGGLLRSGLPWQSVHDGE-Q :
AZC_1310|Azorhizobium_caulinodans_ORS_571 : RARTAGHDLAGRAFLHDYDWRRDDG--FGVLELILTAPVVVASWISLQYYGSTVAPDVFGAGNKLLHNVTGG-IGVVEGNGGLLRAGLPWQSVHDGE-R :
HP15_1623|Marinobacter_adhaerens_HP15 : RSRTRGSNLSGRVFLHDYDPTLDED--GGLLEALLAAPMIVANWINLQYFASVTVPEVYGSGNKLLHSVVGGNVGVVEGNNARLRIGLPLQSVHDGT-H :
CV_3220|Chromobacterium_violaceum_ATCC_12472 : RAKTRKLDLGGRAFLHEYDWRADPD--GKGLQAILAAPMVVAHWINMQYFASTADPRRFGSGNKLLHNVVGGRIGVFEGNSGDLRIGLAWQSVHDGQ-R :
NH8B_2409|Pseudogulbenkiania_sp._NH8B : RARTAQLDLGGRAFLQEYDWRQDAD--ASRLTGILAGPLVVAHWINMQYFASVVDPTRFGSGNKLLHNVVGGRIGVFEGNSGDLRIGLSWQSVHDGS-R :
IL0525|Idiomarina_loihiensis_L2TR : ADRLTSADTIGSNFLHDYNSINDPD--GELLAQLMSAPGLVANWINWQYYCSVTEPKQLGSGNKLLHNRVANDIGVFEGNGGDLRQGLAWQSVHNGS-D :
Mmwyl1_3800|Marinomonas_sp._MWYL1 : RQKTRHLDLEGRSFLHDYDPQNDPD--FAILERILTAPMLVTHWINMQYNLSVTDNFKFGCGNKVLHNAVGGNIGVFEGNGGDLRIGLSMQSLNDGQ-K :
Tcr_0854|Thiomicrospira_crunogena_XCL-2 : RARTRGVDFQGRAFLHDYDWQQDAD--NSLLTLIMTAPMVVTNWINLQYYASVCDNHVYGSGNKVLHNVVDGCIGVFEGNGGDLRIGLPMQSLHNGE-K :
HP15_4110|Marinobacter_adhaerens_HP15 : RSATRHLDLGGRSFLHDYRWREDEG--FNILELIMTAPMVVTHWINLQYFMSVTDNLHYGSGNKVLHNVVGGHLGVFEGNGGDLRIGLPLQSVHDGK-R :
TOL_3715|Thalassolituus_oleivorans_MIL-1 : RQRTRGLNFDGRAFLHDYDSKDDEG--FAVLEQIMTAPMIVTHWINMQYNASVTDPLKFGSGNKVLHNVVGGNLGVFEGNGGDLRIGLSQQSLHDGQ-Q :
VC1582|Vibrio_cholerae_O1_biovar_El_Tor_str._N1696 : RQRSKQAKLDGRVFLHEYQPERDPE--GQLLTQIMTAPMLVTHWINMQYFASTVDNRRFGSGNKTLHNVVGGNIGLFEGNGGDLRCGLALQSLHDGQ-G :
RGE_20630|Rubrivivax_gelatinosus_IL144 : RARSLGAVLDGRVFLHDYDPATDAD--GRQLEQLMTAPMLVAHWINWQYHASTCEPARLGSGNKLLHNVVGGTLGVFEGNGGDLRIGLSRQSLHDGR-R :
Hsero_2320|Herbaspirillum_seropedicae_SmR1 : RARSRGIDLGGRCFLHDYDPTEDGE--GALLEQLMTAPMLVTHWINWQYHASTSDPQRLGSGNKLLHNVVGGAIGVFEGNGGDLRIGLARQSLHDGQ-R :
Acav_3940|Acidovorax_avenae_subsp._avenae_ATCC_198 : RSRTWGTDLGGRAFLHDYDPAQDAD--GSLLEQLMTAPMLVTHWINWQYHASTCDPGRLGSGNKLLHNVVGGHIGVFEGNGGDLRIGLSRQSLHDGQ-R :
Msip34_2093|Methylovorus_glucosetrophus_SIP3-4 : RQRSLGIGLEGRSFLHDYDASQDTD--GSVLELLMTAPMLVTHWINWQYHASTCDPQRLGSGNKLLHNVVGGNLGVFEGNGGDLRIGLSRQSLHDGK-H :
Alide2_2978|Alicycliphilus_denitrificans_K601 : RHRTQGAALGGRSFLHDYDTDLDGD--GSVLELLMTAPMLVTHWINWQYHASTCDPSRLGSGNKVLHNVVGGTLGVFEGNGGDLRIGLSRQSLHDDQ-R :
U875_08155|Pandoraea_pnomenusa_3kgm : RDRSRGVMLDGRSFLHDYDASRDAD--GSVLELLMTAPMLVTHWINWQYHASTCDPLRLGSGNKLLHNVVGGSLGVFEGNGGDLRIGLSQQSLHDGE-R :
Isop_0797|Isosphaera_pallida_ATCC_43644 : RERTRGVNLEGRAFLHEYDAARDPE--QSVLTLILTAPMVVASWINLQYFASTIDQATFGCGDKALHNRVGG-LGVVLGNGGDLRGGLAVQSVHDAQGR :
PB2503_13514|Parvularcula_bermudensis_HTCC2503 : RARTYGMDLEGRAFLHSYDHTTDTE--GGILELIMTAPLIVASWISYQYYASSIDNETFGSGDKTLHNNIGD-IGVLEGASGDLRVGLPIQSVHDGM-R :
RB1129|Rhodopirellula_baltica_SH_1 : RELTRGRSLDGRVFLHSYNQTTDPK--GAVLESILTAPMVVAHWINMQYYASTVDPTLFGSGCKTIHNVVG-QFGVLSGNGGDLQAGLPNQSLGCGL-K :
Plabr_4776|Planctomyces_brasiliensis_DSM_5305 : RERTSSLHLAGRTFLHNYDFRQDQS--GAILELIMTAPMVVTNWINLQYYASTVDNRAYGSGNKTVHNLVGQ-FGILSGNGGDLQTGLPWQSVHDGN-G :
TMO_0417|Tistrella_mobilis_KA081020-065 : RALTRHLDLGGRTFLHSYDWQADAD--GRLLEVILTAPMVVAEWINTQYLFSTLDNDTWGAGSKTIHDPVA-GLGVLKGNGPDLAVGLPLQSVMVADGQ :
MTES_1382|Microbacterium_testaceum_StLB037 : RAITAGHDLGRRAFLHSYEAAADPD--GSGLETILTAPMIVAQWINSQYAASTVAPDRFGAGPKPLHNVVG-TVGVLSGYGGDVRLGLPWQSV-GVGQE :
Micau_3943|Micromonospora_aurantiaca_ATCC_27029 : RELSAGRDLGCRTFLHSYDWTADPD--AVALETIMTGPLVVASWINLQYYFSTVDPHRLGAGTKTVHTVLGDALGVLSGSGGDLRAGLPLQSV-DDGTR :
BA_3182|Bacillus_anthracis_str._Ames : RELTQECDLEGRAFLHNYDWKQDES--GDILANIIAGPGTVAQWINLQYYASTVAPHYYGSGNKATQTVTA-GLGVMQGNASDLLPGLPWQSVMQSDRE :
HD73_2817|Bacillus_thuringiensis_serovar_kurstaki_ : RELTQDCDLEGRAFLHNYDWKQDES--GDILANIIAGPGTVAQWINLQYYASTVAPHYYGSGNKTTQTVTA-GLGVMQGNASDLLPGLPWQSVMQSDSE :
BSU01845|Bacillus_subtilis_subsp._subtilis_str._16 : RELTQDCDLEGRAFLHNYDWKQDES--GELLANIIVGPGTVAQWINLQYYASTVAPHYYGSGNKATQTVTA-GLGVMQGNASDLLAGLPWQSVMESDHE :
SA0412|Staphylococcus_aureus_subsp._aureus_N315 : RQLTKGIDLEGRTFLHNYDWRKDKD--GKLLNTIISGPALVAQWINLQYYASTVAPHFYGSGNKATQTVTS-GVGVMQGNASDLMYGLSWQSVMAADRT :
Theco_3474|Thermobacillus_composti_KWC4 : RHLTESCNLEGRVFLHSYDWREDPD--GTLLMNIAAGPVTVAQWINLQYYASTVAPHIYGSGNKATQTVTA-GIGVMQGNGSDLLAGLPWQSVMASDRE :
Tmar_0818|Thermaerobacter_marianensis_DSM_12885 : RALTRHVHLDGRVFLHSYDWRSDPY--GERLAAIVAGPVTVGQWINLQYYASTVAPHVYGSGSKATQTVTA-GIGVMQGNGSDLMTGLPWQSVAASDRE :
GTCCBUS3UF5_5570|Geobacillus_thermoleovorans_CCB_U : RALTKGVHLDGRVFLHSYDWREDPT--GEALAGIIAGPATVGQWINLQYYASTVAPNYYGSGDKTTQTVTG-GIGVMQGNGSDLLAGLPWQSVAASDRE :
LPE509_03005|Legionella_pneumophila_subsp._pneumop : RQLTKNIDLEGRCFLHSYDWSKDED--GTLLETILTAPMVVAQWINTQYLFSTIDNVAYGSGSKITHNVAG-KIGVMQGNASDLMHGLPLQSVMSHDEK :
Slin_0816|Spirosoma_linguale_DSM_74 : RQLTASLNLQGRSFLHSYNYTQDPS--GSSLTTILTAPMVVAEWINTQYLFSTLDNVAFGGGSKITQNITG-KIGIMQGNGSDLMTGLPLQSVYASDEL :
SNE_A16250|Simkania_negevensis_Z : RKLTVGIDLMGRSFLHSYDWDQDPT--DKILEAILMGPMVVAEWINMQYFFSTLDPLAFGSGSKVTHNVVG-KIGVMQGNGSDLMFGLPLQSVHVNDHT :
Belba_2581|Belliella_baltica_DSM_15883 : RALTKSLNLHGNCFLHSYNWELDTD--GNALAAIMQGPMVVTQWINNHYYFATVDNENYGGGSKITHNVTG-RFGVMQGNGGDLKRGLPLQSVNESDQQ :
P700755_003031|Psychroflexus_torquis_ATCC_700755 : RSLTKTMNLHGNCFLHSYNWKLDTT--GDALEQIMQGPMVVTQWINNHYYFSSVDNETYGGGSKITHNITG-TFGVVQGNGGDLKIGLPLQSVNETDEK :
Aeqsu_2834|Aequorivita_sublithincola_DSM_14238 : RSLTKNLNLDGRYFLHSYNWEQDLE--GAALEAIMQGPMVVTQWINSHYYFSTVDNETFGGGSKITHNITG-KYGVVQGNGGDLKTGLPLESVKQSDTE :
DDD_3132|Nonlabens_dokdonensis_DSW-6 : RSLTKNENLHSRCFLHSYDWQQDIT--GAALEGIMQGPMVVTQWINNHYYFSTVDNKTFGGGTKITQNVTG-RYGVLQGNGGDLKMGLPLQSVSETDSK :
ZPR_2927|Zunongwangia_profunda_SM-A87 : RGLTKGLNLNGRCFLQSYDWKTDKD--GTALSAIMQGPMVVTQWINNHYYFSTVDNEKFGAGSKITHNITG-KFGVMQGNGSDLTKGLPLQSLKRSDSE :
SRM_02272|Salinibacter_ruber_M8 : RALTRGLDLDGRCFLHSYDWATDDD--GTALENIMTGPLVVGEWINTQYYFSTVDNAAYGSGSKVTQNVVG-KLGVVQGNGGDLMSGLPLQSLKADDEH :
Htur_0297|Haloterrigena_turkmenica_DSM_5511 : RELTEDRNLDGRAFLHSYDWTTDPD--GDALEAIFTGPLVVTQWINNQYYFATVDNAVYGSGSKITQNPIG-NVGVLQGNGGDLMTGLPLQSLKVDDDQ :
NJ7G_0232|Natrinema_sp._J7-2 : RELTADRDLDGRAFLHSYDWTTDPD--GDALEAILTGPLVVTQWINNQYYFATVDTGVYGSGSKVTQNPVG-NVGVVQGNGGDLLTGLPLQSLKRDDER :
Halxa_1627|Halopiger_xanaduensis_SH-6 : RELTDDENLDGRAFLHSYDWTTDPE--GDALEAIMTGPLVVTQWINNQYYFATVDNGVYGSGSKVTQNALG-NVGVVQGNGGDLMTGLPLQSLQLSDDE :
Natoc_0177|Natronococcus_occultus_SP4 : RGLTDDEDLDGRAFLHSYDWSTDPD--GDALELIMAGPLVVTQWINNQYYFATVDNDVYGSGSKVTQNPVG-NVGVVQGNGGDLMTGLPLQSLKAADDE :
NP5058A|Natronomonas_pharaonis_DSM_2160 : RELTADSDLDGRAFLHSYDWTTDPD--GDALELIMLGPLVVTQWINNQYYFATVDNRVYGSGSKVTQNPVG-NIGVVQGNGGDLMMGLPLQSLMSDDDS :
Hqrw_2948|Haloquadratum_walsbyi_C23 : RRLTDDVDLEGRVFLHSYDWQQDET--GSALKSILTGPLIVTQWINAQYYFATVDTAVYGSGSKVTQNPVG-NVGIYQGNGGDLMRGLPIQSVRKSTDN :
Hmuk_0931|Halomicrobium_mukohataei_DSM_12286 : RELTDGLDLDARAFLHSYDWTTDDE--GDALEAILTGPMVVTQWINAQYYFSTVDNAVFGSGSKVTHNPVG-NVGVYQGNGGDLLTGLPLQSLMAADDE :
HTIA_0481|Halorhabdus_tiamatea_SARL4B : RELTSDLDLDGRAFLHSYDHTTDPD--GDALAAIFTGPLVVTQWINAQYYFSTVDTAVYGSGSKITQNPVG-NVGVYQGNGGDLMTGLPLQSLKASVDR :
HVO_2410|Haloferax_volcanii_DS2 : RELTSDLDLDGRAFLHSYDHATDPD--GDALEAILTGPMVVTQWINAQYYFSTVDNAVYGSGSKVTQNPVG-NVGVYQGNGGDLMTGLPLQSLMADDDT :
pNG7034|Haloarcula_marismortui_ATCC_43049 : RELTSDVDLDGRAFLHSYDWSTDPD--GEALEAILTGPMVVTQWINTQYYFSTVDNAVYGSGSKVTHNPVG-NVGVYQGNGGDLMTGLPLQSLMAADDD :
Hbor_38520|Halogeometricum_borinquense_DSM_11551 : RELTSGCDLDGRAFLHSYDWSTDPN--GDALEAILTGPMVVTQWINAQYYFSTVDNAVYGSGSKVTQNPVG-NVGVYQGNGGDLMAGLPLQSLMIADRE :
Nham_4323|Nitrobacter_hamburgensis_X14 : RSLSERADLQGRPFLMSYDWRCDPK--GRLLENLLAAAVVVGQWINLEHFFSTVDNARLGSGSKAYHNVAG-RFGVMTGSLSDLRTGLPAQTVMREG-Q :
Nham_4329|Nitrobacter_hamburgensis_X14 : RSLSERADLQGRPFLMSYDWRCDPK--GRLLENLLAAPVVVGQWINLEYFFSTVDNARLGSGSKAYHNVAG-RFGVMTGSLSDLRTGLPAQTVMREG-Q :
THI_0132|Thiomonas_arsenitoxydans : RSLTEAADLEGRPFLQSYDWRCDPK--GRLLENILAAPVVVGEWINLEHFFSTVDNAHMGSGSKAYHNVAG-RFGVMTGNLSDLRTGLPMQTVMREG-R :
Tbd_2653|Thiobacillus_denitrificans_ATCC_25259 : RDATGGLDLEGRVFLHSYDYRCDPR--GRLLENILAGPLVVGQWINMEHYFSAVDNAHYGSGSKVYHNIAG-RFGVMTGNLSDLRTGLPAQTVLKDS-A :
Hneap_0907|Halothiobacillus_neapolitanus_c2 : RHLTEGANLSGRTFLQSYDYRLDPK--GRHLENILSNPLIIGQWINLEHYFSAVDNEHFGSGSKAYHNVVG-RFGVVTGNLSDLRTGLPAQSVLKDG-R :
Marky_1969|Marinithermus_hydrothermalis_DSM_14884 : REVTRALDLKGKVFLHSYDYRVDPK--GRLLENILSGPLIVGHWINMEHYFSTTDNEAYGSGSKVYHNVAG-RIGVMTGNVSDLRTGLPAQTVLKQG-E :
aq_863|Aquifex_aeolicus_VF5 : RELTKHLNLEGKVFLHSYDYRVDRR--GFLLEVILSGPAIVGQWINMEYYFSTTDNEVYGSGSKVYHNVVG-RFGVISGNFSDLRTGLPTQTVYKEG-K :
HTH_1102|Hydrogenobacter_thermophilus_TK-6 : RDITKLSELEGRVFLHSYDYTVDPK--GFLLENILAGPAVVGQWINMEYYFSTTDNEVYGSGSKVYHNVVG-RIGVMTGNYSDLRTGLPAQTVLKEG-K :
Thal_0253|Thermocrinis_albus_DSM_14484 : RSITKLANLDGRVFLHSYDYRVDKK--GFLLENILAGPAVVGQWINSEYYFSTVDNEVYGSGSKVYHNVVG-RIGVMTGNYSDLRTGLPAQTVLKEG-K :
HY04AAS1_0547|Hydrogenobaculum_sp._Y04AAS1 : RSSTQNIALKNRFFMQSYTWEIDKD--NKILKNILSGPFIIGEWINMEHYFSTTDNERLGAGSKVYHNVVA-KVGVWTGNYGDLRTGLPYQTVYHDG-V :
SULAZ_0653|Sulfurihydrogenibium_azorense_Az-Fu1 : SESRINSVLKNRFFLHSYDYKIDVD--NSILKRILNGPLLIAQWINAEHYFSTVDNEKFGSGSKVYHNVVS-RIGVFSGNYSDLKIGLPYQTVYVED-R :
LFML04_2276|Leptospirillum_ferriphilum_ML-04 : RRLTRGIDLGGRVFLQEYEYTLDPS--GKVLETLLSGPLVVARWISLEHYFSTIDNDVYGSGSKVSHNVVG-RFGVQFGNGGDLRMGLPWQTVFDAG-V :
NIDE1770|Candidatus_Nitrospira_defluvii : RELTRPLDLQGRSFLHSYDYRQDAS--GKLLETIMTAPLIVAQWINMEHYFSTVDNEVYGSGSKVYHNIVG-RVGVMSGATSDLRLGLPAQTVL-DGPV :
NIDE4388|Candidatus_Nitrospira_defluvii : REMTKGLNLAGRVFLHSYDYREDPT--DRWLEVLLTAPQVVAQWINMEHYFSAVDNEVYGSGSKIYHNVVG-RIGIMSGPWSDLRLGLAWQTVM-NDDL :
TPY_2995|Sulfobacillus_acidophilus_TPY : RQLTAGHSWGNRVFLHSYDYREDTS--GRWLMNIVNGPLIVAHWINMEYYFSTVDNAVYGSGSKVTANVVG-GLGVMQGSHSDLKPGLPWQSVRDTDGA :
Runsl_0785|Runella_slithyformis_DSM_19594 : RELSKGVFLDRRAFLNSFDYQVDPE--GKYLLNILNAAAPVCGGINLEYFFSRVDNQKLGAGTKLPHNVMG-LFGVANGIDGDLRPGLPSQMIE----- :
Slin_1124|Spirosoma_linguale_DSM_74 : RSLTKGLFLDRRSFLNSYDYRVDPD--GKYLFNILKAAAPVCGGINLEYFFSRVDNQQLGAGTKLPHNVMG-LIGVANGMDGDLRPGLPSQMIE----- :
Halhy_2331|Haliscomenobacter_hydrossis_DSM_1100 : RHLSRGLFLDRRAFLNSYDYRHDPE--GIYLSTILDAAAPVCGGINLEYFFSRVDEQKLGAGSKLPHNVMG-LFGVANGIDGDLRPGLPTQMTE----- :
Emtol_1186|Emticicia_oligotrophica_DSM_17448 : RDLSESLFLDRRSFMNSFDFRVDPE--GKFLVNILNAVAPVCGGINLEYYFSRVDNQKLGAGSKLPHNVMG-LFGVANGIDGDLRPGLPSQMIE----- :
Avin_17870|Azotobacter_vinelandii_DJ : RFLSRKLFLDRRSFLNSYDYRVDPD--GRFLLGILRAAAPVCGGINLEYFFSHVDNQKLGAGSKLPHNVMG-LIGVANGNDGDLRPGLPSQMIE----- :
Nmul_A0708|Nitrosospira_multiformis_ATCC_25196 : RSITENLFLDRRASTSTYDYRTDPE--GKFLTMSMGPIALVMGGIDLEYFFSRTDNHKMGAGTKLPHNVMG-LIGVANGADGDLRTGLPSQMIE----- :
Halhy_3199|Haliscomenobacter_hydrossis_DSM_1100 : RDLTKGLFLDRRAFLNSYDYRTDRE--GKLLPGIMRPLGPVCGGINLEYYFSRVDNYKLGAGTKLPHNVMG-LFGVANSSDGDLRPGLPVQMIE----- :
P700755_000908|Psychroflexus_torquis_ATCC_700755 : RSLTKSVFLDQRAFLNSYDYKQDLN--SIQLKSILQAATPVCGGINLEYYFSSVDNEKLGAGSKLPHNVIG-LFGVANGIKGDLRPGLPSQMID----- :
Metme_3843|Methylomonas_methanica_MC09 : RSLTRHLFMDRRAFLQSYDPNIDQD--GSILVRVLSAAIPVCGGINLEYLFSRIDNSVYGAGTKLPHNVIG-LLGVANGVEGDLRTGLPSQMIE----- :
Turpa_2600|Turneriella_parva_DSM_21527 : RSLTENLFLDRRTFFNSYNPHSDPG--GEILSQILTPFVPVCAGINLAYYFSLLDNSVYGAGSKLPHNIFG-LIGVGNGVDGDLRSGLPEQMIE----- :
STAUR_4541|Stigmatella_aurantiaca_DW4 : RELTAQRFLDRRSFLVSYDPTQDQG--GAQMRSAILGTVPVAVNIAMDYYFSRVDNEGFGAGSKLPLNVVS-LLGVLTGSKSDLRIGLARQMVE----- :
Metme_0526|Methylomonas_methanica_MC09 : RSLTQGAFFDRRLFLISYDPTQDPE--GTILEGILLAVGPVGAGINLEYYFSTVNNERLGCGSKVPHNLTG-FCAVMEGAGSDLRTGLPKQMIE----- :
Nhal_2896|Nitrosococcus_halophilus_Nc_4 : RYVTRGAFFDRRVFLVSYDPTQDPD--GKILENTLMTQGVVGVGINLEYYFSTVDNERFGCGSKVPHNVTG-HFAVMEGASSDLRTGLPRQMIE----- :
MCA0512|Methylococcus_capsulatus_str._Bath : RSVSRGLFLDRRVFLISYDPTRDPE--GRILEGILLAAGPVGAGINLEYYFSTVNNERHGCGSKVAHNVTG-LFGIMEGAASDLRTGLPRQMAE----- :
Nit79A3_1613|Nitrosomonas_sp._Is79A3 : RSVSQGTFLDRRVFLISYDPTQDAD--GKLLENLLLAVSPVGAGINLEYYFSTVNNDFFGSGSKVTHNITG-MFGVLEGTSSDLRTGLTKQMVE----- :
CAP2UW1_3861|Candidatus_Accumulibacter_phosphatis_ : RTLSRGAFFDRRVFLISYDPLPDVE--GRVLEATLLAAGPVGAGINLEYYFSTVNNEHFGCGSKVMHNLAG-LFGVMQGASSDLRTGLPLQMVE----- :
Daro_0565|Dechloromonas_aromatica_RCB : RQMSRGLFLDRRVFLISYDPVGDDD--GCIVEGILLAAGPVGAGIALEYYFSTVDNERFGCGSKITHNITG-LFGVMEGADSDLRTGLPWQMVE----- :
SCE1572_25475|Sorangium_cellulosum_So0157-2 : RELTHGMFLDRRAFLVSYDPTQDPT--GAILERVLLSAGPVGAGINLEYYFSRVDNLRYGAGTKLPHNLAS-LLGVMDGSLSDLRTGLPKQMIE----- :
SCE1572_28620|Sorangium_cellulosum_So0157-2 : RALTRGLFLDRRAFLVSYDPTQDPS--GAILERVLLAVGPVGAGINLEYYFSCVDNRRYGAGTKLPHNLVS-LLGVMEGSLSDLRTGLPKQMIE----- :
Plabr_3227|Planctomyces_brasiliensis_DSM_5305 : RDWSRGLYLDRRAFLTTYDPSVDDEQ-HSILLRILSAAIPVCAGINLEYYFSKVDHTVYGAGSKLPHNLAA-LLGVMEGTSSDLRTGLYQQMVE----- :
RB10314|Rhodopirellula_baltica_SH_1 : RDWSRGLFMDRRAFVTEYDPTVDDEN-GHILTRILQAAIPVCGGISLEYYFSTVDVEGYGCGSKLPHNVAS-MVGVMTGAASDLRPGLSQQMVE----- :
Sinac_5055|Singulisphaera_acidiphila_DSM_18658 : REWSRGLFLDRRAFLTSYDPTEDDAE-CTILTRILQAVFPVCGGINLEYYFSHVDNNGYGAGSKLPHNLAA-LLGVMDGAASDLRTGLPWQMVE----- :
Psta_3079|Pirellula_staleyi_DSM_6068 : RSRVRNLYMDRRCFMHSYDPNSDDAS-SSILGRILAPVVPVCQGINLMYYFSAVDPVGWGSGTKLPHNVTS-LLGVMDGYASDLRTGLPVQGVE----- :
Franean1_5483|Frankia_sp._EAN1pec : RSRTRGLYLDRRSFLVSYDPTADPD--GAVLTRLLLSAAPVGAGINLEYYFSRIDPIGYGAGSKLPHNITG-LVGVMDGHGSDLRTGMPWQSVE----- :
Hoch_4410|Haliangium_ochraceum_DSM_14365 : RERTRGLFLDRRAFLVSYDPEKDDD--GALLGPLLQSVGPVGAGINLEYYFSFVDNARYGAGTKLPHNITG-LIGVMDGHMSDLRTGLSAQMVE----- :
BN6_40270|Saccharothrix_espanaensis_DSM_44229 : RSLTRGLYLDRRAFLASYEPAADTD--GGLLAALLGAVGPVCAGINLEYYFSFVDPAGYGCGTKLPHNITG-LIGVMDGHASDLRTGLPWQMVE----- :
Tcur_2598|Thermomonospora_curvata_DSM_43183 : RSRTRGLFLDRRAFLVSYDPGGDPT--GELLADLLAAVGPVCAGINLEYYFSRIDPAGYGCGTKLPHNIAG-LLGVMDGHASDLRTGLPWQMVE----- :

BLOCKS : BBBBBBBBBBBBBBBBBBBBBBBBBBBBBBBBBBBBBB :
SITE : --X---X------------------------------- :
LFML04_1811|Leptospirillum_ferriphilum_ML-04 : WIHEPLRLNVVIEAPQEAIDDVIARHTLVRDLIENEWL :
LFML04_1818|Leptospirillum_ferriphilum_ML-04 : WIHEPLRLNVVIEAPQEAIDDVIARHTLVRDLIENEWL :
AFE_1661|Acidithiobacillus_ferrooxidans_ATCC_23270 : WIHEPVRLNVVIEAPQAEMESIISRHILVRELVDNGWL :
Afer_0133|Acidimicrobium_ferrooxidans_DSM_10331 : WLHEPVRLTVVVEAPRGAIDQVVREHQLVADLVEHGWL :
Thimo_2946|Thioflavicoccus_mobilis_8321 : WVHEPLRLSVFIEAPEAPIDAIIARHDLVRQLVDNGWL :
THI_0793|Thiomonas_arsenitoxydans : WMHEPLRLSVYLEAPAEAIDNIIARHEMIRQLVDNKWL :
Hneap_0211|Halothiobacillus_neapolitanus_c2 : WRHEPLRLSAYIEAPIAEIDKIIAGHDMLNALINNRWM :
Ftrac_3394|Marivirga_tractuosa_DSM_4126 : YQHEPLRLSVVINAPKEAMIKVLEKHDSVRQLVDNQWI :
S58_63940|Bradyrhizobium_oligotrophicum_S58 : FIHEPVRLNVFIAAPESAMDEIMQRHEGVRDLVGNGWV :
Cagg_2891|Chloroflexus_aggregans_DSM_9485 : YVHEPIRLHVLIEAPIEAMTAIITKHEQVQQLLDNDWL :
RoseRS_3853|Roseiflexus_sp._RS-1 : YVHEPMRLHVMIEAPIDAMTAIIARHEQVRQLLDNGWL :
SPICUR_00740|Spiribacter_sp._UAH-SP71 : RRHDPLRLTVAIQAPKAAISDTLARHPSVQALFDNRWL :
Dshi_0997|Dinoroseobacter_shibae_DFL_12__DSM_16493 : FVHDPLRLTIVVNAPQEAITDILARHDGVRALFDNGWL :
Gal_04259|Phaeobacter_gallaeciensis_DSM_26640 : LQHDPLRLTVVIEAPREAMTQILERHSQVRDLFDNGWL :
Jann_1258|Jannaschia_sp._CCS1 : YAHDPLRLSVCIEAPREAMTDILRRHDGVRALFDNRWL :
RLO149_c003320|Roseobacter_litoralis_Och_149 : FAHEPLRLSVCIEAPREAMSDILKRHDGVRALFDNRWL :
ACMV_25640|Acidiphilium_multivorum_AIU301 : YAHDPLRLSVCIEAPREAMSEILGRHADVRALFDNGWL :
RPA2996|Rhodopseudomonas_palustris_CGA009 : LVHQPLRLSVLIEAPHEAISTILDRYPEVRALFDNRWL :
PXO_00907|Xanthomonas_oryzae_pv._oryzae_PXO99A : LIHEPLRLSVLIEAPTEAIGAILERHPQLRALFDNRWL :
RC1_1188|Rhodospirillum_centenum_SW : LAHEPLRLSVLIEAPREAIAGILERHPGVRTLFDNRWL :
AZC_1310|Azorhizobium_caulinodans_ORS_571 : LTHEPLRLSVLIEAPREAIARILERHPEVRALFDNLWL :
HP15_1623|Marinobacter_adhaerens_HP15 : WRHEPVRLTVLIDAPGDRIESVLRRQPDVAALVENQWV :
CV_3220|Chromobacterium_violaceum_ATCC_12472 : LRHAPLRLAACIDAPPDRLAEALAAQPVPRQLAENGWL :
NH8B_2409|Pseudogulbenkiania_sp._NH8B : WRHEPLRLAACIDAPAVLIEQALARQPDVARLVANRWL :
IL0525|Idiomarina_loihiensis_L2TR : FVHRPMRLQVIVEADEATIQRALAKAVAFNELFEQQWI :
Mmwyl1_3800|Marinomonas_sp._MWYL1 : WMHTPVRLAVYVTAPKSAIEKIAAKHDIVKHLIDNDWL :
Tcr_0854|Thiomicrospira_crunogena_XCL-2 : WMHEPLRLSVYIDAPQKTIAQVVAENDVVRHLIDNEWL :
HP15_4110|Marinobacter_adhaerens_HP15 : WVHEPLRLSVYLAAPREAIAEIAQKHKVVQELIDNDWL :
TOL_3715|Thalassolituus_oleivorans_MIL-1 : WVHEPLRLSVYIQAPQEAIASIISQHDNVRQLVDNQWL :
VC1582|Vibrio_cholerae_O1_biovar_El_Tor_str._N1696 : WRHEALRLTVVIDAPRERIEQVMASHRVVEHLVKHEWL :
RGE_20630|Rubrivivax_gelatinosus_IL144 : WVHEPLRLTVIVDAPAAAIERVIAQHATVHQLVEHGWL :
Hsero_2320|Herbaspirillum_seropedicae_SmR1 : WLHEPLRLTVVIDAPEAAIERVIAAHAVVRQLLENGWL :
Acav_3940|Acidovorax_avenae_subsp._avenae_ATCC_198 : WVHEPLRLTVVIDAPAAAIESVIARHAVLQQLLGNGWL :
Msip34_2093|Methylovorus_glucosetrophus_SIP3-4 : WIHEPLRLTVVIEAPQAAIEAVIAKHAVVKQLVDNGWL :
Alide2_2978|Alicycliphilus_denitrificans_K601 : WVHEPLRLTVIIDAPQAAIDAVIAKHAVVRQLLDNGWL :
U875_08155|Pandoraea_pnomenusa_3kgm : WIHEPVRLTVVVDAPRAAIETVIGKHEVVRHLLDNGWL :
Isop_0797|Isosphaera_pallida_ATCC_43644 : WFHEPLRLQVVVEAPREKIERVLAAHPSVYDLVRGGWV :
PB2503_13514|Parvularcula_bermudensis_HTCC2503 : LRHEPTRLRALIEAPTEAIDAVFDKHPDVKALFDHGWL :
RB1129|Rhodopirellula_baltica_SH_1 : MQHLPLRLQTVVVASRESIDRVIAKHANIRNLLQNGWV :
Plabr_4776|Planctomyces_brasiliensis_DSM_5305 : YQHEPLRLTVVVEAPCEMISKVIEKHESVRHLVSNGWL :
TMO_0417|Tistrella_mobilis_KA081020-065 : IRHEPLRLMAVVQAPRTRVEAAIERHAVLSTLFDNGWV :
MTES_1382|Microbacterium_testaceum_StLB037 : ARHEPVRLQVFVQAPLARVNDIVDSSEVVRTLVANRWI :
Micau_3943|Micromonospora_aurantiaca_ATCC_27029 : PAHDPLRLLAVVHAPHHLVDTVLGRNPALRQLIDGGWM :
BA_3182|Bacillus_anthracis_str._Ames : TYHSPLRLLIVIQAPTKYIERLLNNNFTFREKVQNGWV :
HD73_2817|Bacillus_thuringiensis_serovar_kurstaki_ : TYHSPLRLLIVIQAPIEYIERLLNNDFTFREKVQNGWV :
BSU01845|Bacillus_subtilis_subsp._subtilis_str._16 : AYHSPLRLLILIQAPREYVERLLNHDSAFLQKVQNGWV :
SA0412|Staphylococcus_aureus_subsp._aureus_N315 : MYHSPIRLLVVIQAPDYVVARLFANNEHFARKVSNHWL :
Theco_3474|Thermobacillus_composti_KWC4 : WFHSPLRLLVVIEAPRPYMVKLLEGNPEFRRKVSNGWL :
Tmar_0818|Thermaerobacter_marianensis_DSM_12885 : VVHAPLRLLVVIEAPREWIQRLLQRDAQFRQKVRHGWI :
GTCCBUS3UF5_5570|Geobacillus_thermoleovorans_CCB_U : WFHSPLRLLVIIEAPSSYIERLLDENSEFRRKVQNGWL :
LPE509_03005|Legionella_pneumophila_subsp._pneumop : SFHEPQRLLTVVYAPREIISELVEKHDVLKTLFFNEWV :
Slin_0816|Spirosoma_linguale_DSM_74 : AYHQPQRLLTVVYAPRPLLDAIIQAQPVLQKLFGNGWV :
SNE_A16250|Simkania_negevensis_Z : PYHELQRLITIIYSPPSKISRILEKQQVLRKLFLNQWV :
Belba_2581|Belliella_baltica_DSM_15883 : YYHQPLRLSVYIHAPIARVTEILEKYNHLKSLITNEWI :
P700755_003031|Psychroflexus_torquis_ATCC_700755 : AYHQPLRLSVLIQAPKQRIQEILNKNKNLKSLLDNEWI :
Aeqsu_2834|Aequorivita_sublithincola_DSM_14238 : MYHQPLRLSVIIQAPIENIENILNKNENLKSLMNNEWI :
DDD_3132|Nonlabens_dokdonensis_DSW-6 : LYHQPLRLSVLINAPLPKVIDILSRNAHLQTLLDNEWI :
ZPR_2927|Zunongwangia_profunda_SM-A87 : MYHQPLRLSVVIETPTNRVEKILEEQPHLKNLLDNEWI :
SRM_02272|Salinibacter_ruber_M8 : VHHRPLRLMALIQAPTDRVEAILDRHAAVAHLFDHEWM :
Htur_0297|Haloterrigena_turkmenica_DSM_5511 : PYHQPLRLTAVIHAPVDRVTEILRKHGDVRTLLDNGWI :
NJ7G_0232|Natrinema_sp._J7-2 : PFHQPLRLTAVIHAPVERVTEILRDHEDVRELLDNGWI :
Halxa_1627|Halopiger_xanaduensis_SH-6 : SYHQPLRLTAVIHAPVERVTDILQRHDEVAELLDNGWI :
Natoc_0177|Natronococcus_occultus_SP4 : PYHQPLRLTAVIHAPVERVTEILREHENVERLVANGWI :
NP5058A|Natronomonas_pharaonis_DSM_2160 : PYHQPLRLTAVIHASVENVTDILREHGHVRRLVDNGWV :
Hqrw_2948|Haloquadratum_walsbyi_C23 : LYHQPIRLSTVVHAPVSKVTHALADLESVTELLDNNWI :
Hmuk_0931|Halomicrobium_mukohataei_DSM_12286 : PYHQPLRLSTVVHAPVERVTDVLADNPEVAELLDNDWL :
HTIA_0481|Halorhabdus_tiamatea_SARL4B : PHHQPLRLSVAVHAPVERVTDVLAENPAVTELLDNDWL :
HVO_2410|Haloferax_volcanii_DS2 : PYHQPLRLSTIIHAPVDRVTDVLADHAELTELLDNDWL :
pNG7034|Haloarcula_marismortui_ATCC_43049 : PHHQPLRLSTVIHAPVDRVTDVLADHAELATLLDNNWL :
Hbor_38520|Halogeometricum_borinquense_DSM_11551 : PYHQPLRLSTVVHAPVERITDILADHEELTEILDNNWL :
Nham_4323|Nitrobacter_hamburgensis_X14 : PYHEPMRMIALIEAPLDFAGRALQSVVKVKSLVLGGWI :
Nham_4329|Nitrobacter_hamburgensis_X14 : PYHEPMRMIALIEAPLDFAGRALQSVVKVKSLVLGGWI :
THI_0132|Thiomonas_arsenitoxydans : PYHEPMRLIALIEAPLDFAGRALQSVVKVKSLVLGGWI :
Tbd_2653|Thiobacillus_denitrificans_ATCC_25259 : PYHEPLRLLTVIEAPFAHARAAVEGVVKVKNLMHNGWL :
Hneap_0907|Halothiobacillus_neapolitanus_c2 : PYHEPIRLLAIIEAPAAFTLEVAGRLPKVMSLITNGWI :
Marky_1969|Marinithermus_hydrothermalis_DSM_14884 : PFHLPVRLIVLLEAPFAFARTAIERVHKVRELMHKGWL :
aq_863|Aquifex_aeolicus_VF5 : PLHIPARLIILLEAPYEFALSVINRVYAIRNLIQNEWV :
HTH_1102|Hydrogenobacter_thermophilus_TK-6 : PFHVPVRYTLVIEAPLELSQRAISRIRKIRDLMQNEWI :
Thal_0253|Thermocrinis_albus_DSM_14484 : PFHIPIRYTLIVEAPFELARNAINKIRKIRDLMQNGWI :
HY04AAS1_0547|Hydrogenobaculum_sp._Y04AAS1 : PYHEPIRLLTFIEAPAEKVLEAAQEVKEALKLVVNEWV :
SULAZ_0653|Sulfurihydrogenibium_azorense_Az-Fu1 : PFHEPIRLIAFVEAPLEKVVEAASQTDHPMMLVKNEWI :
LFML04_2276|Leptospirillum_ferriphilum_ML-04 : SRHEPMRLLCVICAPREKVESVLGGNLALSGPFDRGWA :
NIDE1770|Candidatus_Nitrospira_defluvii : PYHEPMRLLTVIEAPRALVESVIAKHPGLERLFQNEWV :
NIDE4388|Candidatus_Nitrospira_defluvii : PYHEPMRLLTLVEASRPRIEKLIARHDVLQHFYHNEWV :
TPY_2995|Sulfobacillus_acidophilus_TPY : PYHEPMRLLVVIEAPRERIQGVLDALPLFRQLVHNAWI :
Runsl_0785|Runella_slithyformis_DSM_19594 : -VHDPVRLLMIVEHFPEVVLTTIQKNPATYEWFINEWV :
Slin_1124|Spirosoma_linguale_DSM_74 : -VHDPVRMFFIIEQFPDVVLDVIKQSSATYEWFINEWV :
Halhy_2331|Haliscomenobacter_hydrossis_DSM_1100 : -VHDPLRLLMIVEHDPVVVLKSLLRSAATYEWFINEWI :
Emtol_1186|Emticicia_oligotrophica_DSM_17448 : -VHDPIRLLVIVEHFTDIVLSAIQKSAQTYEWFFNEWI :
Avin_17870|Azotobacter_vinelandii_DJ : -VHHPVRMMIVVEHFPAVVLNTIRQQPATWEWFANEWL :
Nmul_A0708|Nitrosospira_multiformis_ATCC_25196 : -IHDPVRLLVIIEHYPDVALKVIKSQTANYSFYENYWV :
Halhy_3199|Haliscomenobacter_hydrossis_DSM_1100 : -VHDPVRLLIIVEHFPEVVLKTIQSSPEMYEWFINEWV :
P700755_000908|Psychroflexus_torquis_ATCC_700755 : -VHAPLRLMMIIEHYPEVVMQLIQNDTELWNWYKNEWV :
Metme_3843|Methylomonas_methanica_MC09 : -VHEPARLLLVVEQRIEVVDRALAKLGSLLEWLENDWV :
Turpa_2600|Turneriella_parva_DSM_21527 : -IHDPFRLLIIIEQSREIVERVLASNLSVAGWIQKDWV :
STAUR_4541|Stigmatella_aurantiaca_DW4 : -LHEPMRILVLVEAAEKDLRNLIETHPRMRRMVQGQWM :
Metme_0526|Methylomonas_methanica_MC09 : -IHEAMRLQIVVEAKTTVLEKIYNRQESLRELIGGGWV :
Nhal_2896|Nitrosococcus_halophilus_Nc_4 : -IHEAMRLLTVVESTPEMLTEIYKRQTMIQELVGGGWL :
MCA0512|Methylococcus_capsulatus_str._Bath : -VHEAMRLQVVVEQTPEILAAIYRRQPPIRELVGNGWI :
Nit79A3_1613|Nitrosomonas_sp._Is79A3 : -IHEPLRLQVLVEAKTEILGQIYERQASIRELVGGGWI :
CAP2UW1_3861|Candidatus_Accumulibacter_phosphatis_ : -VHEPMRLLVIVEQTLEIVTAVYQRQPPLQELIGNGWI :
Daro_0565|Dechloromonas_aromatica_RCB : -IHEPMRLLVVVEQTPEVLTAIVGRQPPLQELINNEWI :
SCE1572_25475|Sorangium_cellulosum_So0157-2 : -IHEPVRMLTVVEASAATAAAICARQPALRELIVNGWI :
SCE1572_28620|Sorangium_cellulosum_So0157-2 : -IHEPVRLLVVVEASTDTAAALCARQPALRELICNGWI :
Plabr_3227|Planctomyces_brasiliensis_DSM_5305 : -IHEPLRMTFVIETTPEAMLSIMERVPAIGRLCRGEWV :
RB10314|Rhodopirellula_baltica_SH_1 : -IHEPMRILFVIETTPEMLKKIISENEGIRRMVEGNWV :
Sinac_5055|Singulisphaera_acidiphila_DSM_18658 : -IHEPVRSLFIIETTPESMLRIIERNEGIGRLCRNGWI :
Psta_3079|Pirellula_staleyi_DSM_6068 : -IHEPMRLLFVIETTPERILAIMDRDPVVGRILKNGWA :
Franean1_5483|Frankia_sp._EAN1pec : -IHEPMRLLVIAEAEPERLARIVRENPPLRGLVEGGWI :
Hoch_4410|Haliangium_ochraceum_DSM_14365 : -IHEPVRLLNIVEAEFDVLGRVMERHPVVANLVQNGWI :
BN6_40270|Saccharothrix_espanaensis_DSM_44229 : -IHEPVRLLLVVEASPERLSAIVAADPALERLVSGKWI :
Tcur_2598|Thermomonospora_curvata_DSM_43183 : -IHEPLRLLLVVEAAPQRLEAILRTRPDLGRLVTNGWI :
